# Enhancing prime editing efficiency by modified pegRNA with RNA G-quadruplexes

**DOI:** 10.1093/jmcb/mjac022

**Published:** 2022-04-12

**Authors:** Xiangyang Li, Xin Wang, Wenjun Sun, Shisheng Huang, Mingtian Zhong, Yuan Yao, Quanjiang Ji, Xingxu Huang

**Affiliations:** Gene Editing Center, School of Life Science and Technology, ShanghaiTech University, Shanghai 201210, China; University of Chinese Academy of Sciences, Beijing 100049, China; Gene Editing Center, School of Life Science and Technology, ShanghaiTech University, Shanghai 201210, China; University of Chinese Academy of Sciences, Beijing 100049, China; Gene Editing Center, School of Life Science and Technology, ShanghaiTech University, Shanghai 201210, China; University of Chinese Academy of Sciences, Beijing 100049, China; Gene Editing Center, School of Life Science and Technology, ShanghaiTech University, Shanghai 201210, China; University of Chinese Academy of Sciences, Beijing 100049, China; Institute for Brain Research and Rehabilitation, Guangdong Key Laboratory of Mental Health and Cognitive Science, Center for Studies of Psychological Application, South China Normal University, Guangzhou 510631, China; Hangzhou Global Scientific and Technological Innovation Center, Zhejiang University, Hangzhou 311215, China; School of Physical Science and Technology, ShanghaiTech University, Shanghai 201210, China; Gene Editing Center, School of Life Science and Technology, ShanghaiTech University, Shanghai 201210, China; Guangzhou Laboratory, Guangzhou International Bio Island, Guangzhou 510005, China

**Dear Editor**,

Recent study shows that the prime editing system fusing the Cas9 nickase and reverse transcriptase could perform all types of gene modifications, including base substitutions (transitions and transversions), small insertions, and deletions, without requiring donor DNA or double-strand breaks (DSBs) (Anzalone et al., 2019). Despite the accuracy and versatility, the efficiency of the prime editor (PE) is often insufficient, which limits its broad applications.

In principle, the special prime editing guide RNA (pegRNA) of the prime editing system contains an extra reverse transcription (RT) template and a primer binding site (PBS) in the 3′ extension of the scaffold (Anzalone et al., 2019). Compared with original single guide RNA, pegRNA harbors an extra 3′ extension (RT and PBS) of the scaffold and the 3′ non-structured terminal is more prone to being degraded in cells (Houseley and Tollervey, 2009; Supplementary Figure S1A). Previous reports have demonstrated that forming a special motif at the 3′ end of sgRNA can improve the stability of CRISPR/Cas9 and target cleavage efficiency (Nahar et al., 2018). Recently, we demonstrated that incorporating structured RNA motifs to the 3′ terminus of pegRNAs enhances the efficiency of prime editing (Liu et al., 2021). Therefore, we reason that adding a special motif-based rational structure for the 3′ end of pegRNA may improve the stability of pegRNA, and thus improve the PE efficiency.

We designed three special types of modifications to improve the stability of pegRNA (Figure 1A). One is a natural G-quadruplex (Supplementary Figure S1D), a noncanonical secondary structure formed by G-rich stretches of nucleic acids, the center of which contains a monovalent cation (potassium or sodium) that stabilizes the quartet (Pandey et al., 2013). The second is a non-structured modification with a mutant G-quadruplex sequence to block its function and form a non-structured 3′ extension to protect the functional region of pegRNAs (Supplementary Figure S1B). The third is a hairpin-stem modification, which is the most common secondary structure found in almost every RNA folding prediction, and consists of a double-stranded RNA stem. Compared with the non-structured modification, the hairpin-stem may be a more rational modification to stabilize the 3′ end of pegRNAs (Supplementary Figure S1C). Meanwhile, to keep the length of the pegRNA 3′ extension as small as possible, we select multiple types of three-G-quadruplex motifs (G_3_N_1–3_G_3_N_1–3_G_3_N_1–3_G_3_) that come from different genes (Zhang et al., 2011) and set the lengths of the hairpin-stem and non-structure motifs as 17 nt and 18 nt, respectively (Supplementary Figure S1E).

**Figure 1:**
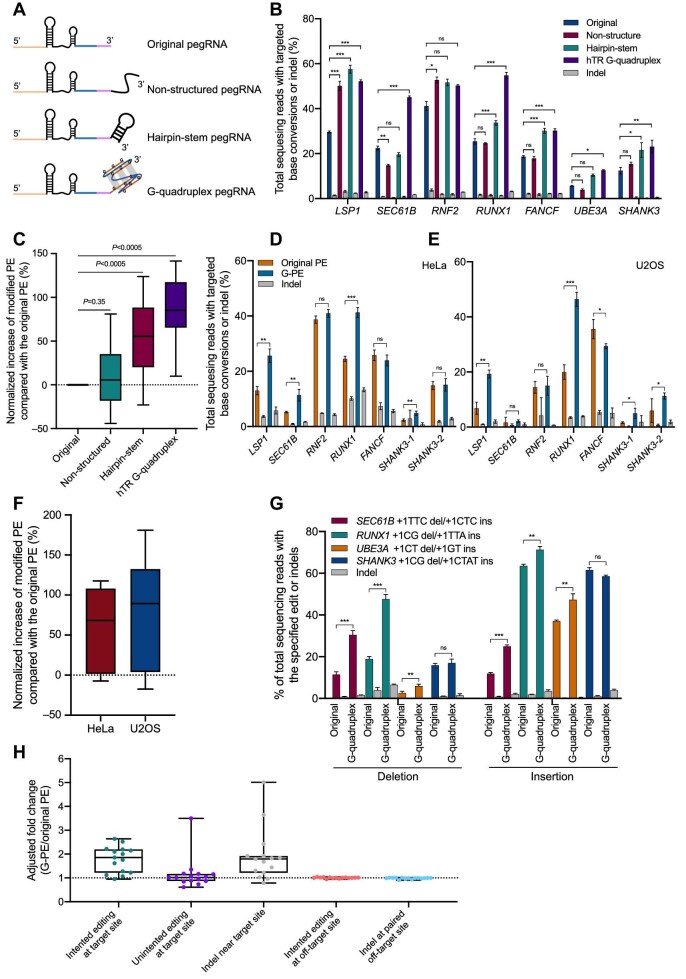
G-quadruplex-modified pegRNA enhances prime editing efficiency at endogenous target sites. (**A**) A schematic of pegRNA with 3′ extended modification. An original pegRNA consists of spacer (orange), scaffold (black), RT (blue), and PBS (purple). The structured RNA modification is fused to the 3′ terminal of pegRNA, forming non-structured pegRNA, hairpin-stem pegRNA, and G-quadruplex pegRNA. (**B**) G-quadruplex-modified pegRNA observably enhances prime editing activity at endogenous target sites in HEK293T cells. PCR amplicons from the target regions were analyzed by targeted deep sequencing. The reads only harboring correct edit were counted to evaluate the editing efficiency, and the reads harboring any unintended insertion or deletion were counted to evaluate the indel frequency. The gray bar indicates the indel frequency coupled with the editing efficiency indicated by the left closest bar. (**C**) Statistical analysis of normalized increase of targeted base conversions in **B**. Means from three independent experiments were used for analysis. (**D** and **E**) Comparison of the prime editing efficiencies of the original PE and G-PE at endogenous sites in HeLa and U2OS cells. The gray bar indicates the indel frequency coupled with the editing efficiency indicated by the left closest bar. (**F**) Statistical analysis of normalized increase of targeted base conversions in **D** and **E** (HeLa and U2OS cells). Means from three independent experiments were used for analysis. (**G**) Increasing targeted deletion and insertion efficiency by G-PE in HEK293T cells. The gray bar indicates the indel frequency coupled with the editing efficiency indicated by the left closest bar. (**H**) The correct editing, unintended editing, and indel frequencies induced by G-PE normalized to those of the original PE at 15 endogenous targets. The off-target editing frequencies of pegRNA and nick sgRNA of G-PE normalized to those of the original PE at three endogenous targets in HEK293T cells. The analysis of adjusted fold increase is described in the Supplementary Materials and methods. Mean ± SD, *n* = 3 independent experiments (*P < 0.05, **P < 0.005, ***P < 0.0005).

We first checked the effects of the G-quadruplex on the PE efficiency at endogenous target sites in HEK293T cells. We designed and constructed paired sgRNA (mCherry signal), pegRNA (GFP signal), and G-quadruplex-modified pegRNA (GFP signal) plasmids, respectively (Liu et al., 2020). The PE, together with pegRNA and paired sgRNA, was transfected into HEK293T cells with EZ Trans Reagent. The GFP–mCherry double-positive cells were harvested and sorted for efficiency analysis 3 days post-transfection. We generated base conversions with PE using modified and original pegRNAs at seven sites, including *RNF2, RUNX1, LSP1, SEC61B, UBE3A, FANCF*, and *SHANK3* (Supplementary Figure S2A). We observed increased editing efficiency at 6 out of 7 sites (85.7%) by human telomerase RNA (hTR) G-quadruplex modification and 3 out of 7 sites (42.8%) by hairpin-stem modification, but no effects by non-structure-modified pegRNAs (Figure 1B). Compared with original pegRNAs, hTR G-quadruplex modification gave >80%, while hairpin-stem modification gave ∼50% increase in the editing of endogenous targets (Figure 1C). Interestingly, non-structure-modified pegRNA with a longer 3′ extension, which originates from mutated hTR G-quadruplex, had no effects on the PE efficiency, indicating that the specific structure of the 3′ extension is critical for the improvement. To further explore the effects of G-quadruplex modification, three other types of G-quadruplexes that originated from *MT3, TERRA*, and *NRAS* genes were also appended to the 3′ end of pegRNAs. The results showed that *MT3* and *NRAS* G-quadruplex-modified pegRNAs induced significant increase in the editing efficiency of endogenous sites compared with original pegRNAs, while the *TERRA* G-quadruplex-modified pegRNA performed inconsistently (Supplementary Figure S2B). The *MT3* and *NRAS* G-quadruplex modifications induced a median of ∼25% and ∼60% increase in the PE efficiency, respectively (Supplementary Figure S2C and D). Overall, the secondary structural modifications, especially the hTR G-quadruplex, at the 3′ end of pegRNAs can significantly increase the editing efficiency of PE at endogenous target sites.

Next, we further characterized the effects of G-quadruplex modification of the 3′ extension using hTR G-quadruplex modification, hereafter G-PE. The G-quadruplex was also a structured RNA but shorter than evopreQ_1_, mpknot, and xrRNA described in a recent study (Nelson et al., 2022). Then, we compared the editing activity of G-PE with these three modifications at three endogenous sites in HEK293T cells. The results showed that G-PE performed comparable editing activity with the PEs of three modifications (Supplementary Figure S3A and B). Furthermore, we compared the editing efficiency of G-PE with evopreQ_1_ modification at four sites reported in a recent study (Nelson et al., 2022) and demonstrated that G-PE generated a similar editing level to evopreQ_1_ (Supplementary Figure S3C). Considering that the length of PBS and RT template usually affects the editing activity of PE (Anzalone et al., 2019), we tested the activity of G-PE to mediate targeted editing at four endogenous sites with different combinations of PBS and RT template. Compared with the original PE, the editing efficiency of G-PE at each site showed an almost comparable increasing level at different combinations of PBS and RT template, and we found that the combination of PBS and RT template did not affect the ability of G-quadruplex to improve the PE efficiency (Supplementary Figure S4A–D).

Further, we tested the base conversion efficiency of G-PE at the same seven endogenous sites in different cell lines, including HeLa and U2OS cells, respectively (Supplementary Figure S5A and B). Similarly, G-PE induced 1.7- and 1.9-fold higher editing efficiency than the original PE at 4 out of 7 and 3 out of 7 target sites in HeLa and U2OS cells, respectively (Figure 1D–F). We also tested the editing of the original PE and G-PE at four endogenous sites without sorting. We seeded HEK293T cells into a 96-well plate and transfected cells with Lipofectamine 2000 reagent. Then, all cells of each well were harvested for sequencing 3 days after transfection (Supplementary Figure S6A). The results showed that G-PE performed improved editing, similar to sorted samples (Supplementary Figure S6B and C). Other than base substitutions, PE also could induce precise insertion and deletion (indel) without DSBs (Anzalone et al., 2019). Therefore, we asked whether G-PE could increase precise indel compared with PE in HEK293T cells. Then, we designed four sites for precise deletion and insertion, ranging from −3 bp to +4 bp. The results showed that G-PE induced precise indel by an increased editing efficiency at 3 out of 4 sites compared with the original PE (Figure 1G). It is worth noting that G-PE increases precise indel at the same sites in HeLa cells (Supplementary Figure S5C).

We also explored whether G-PE could induce a high-fidelity genome editing. We firstly analyzed 15 target sites to comprehensively evaluate the level of unintended edits, including mutation, deletion, and insertion, at the endogenous target sequences and found that almost all sites had <1% of unintended editing efficiency (Supplementary Figure S7A). To further compare the frequencies of unintended editing, we adopted adjusted fold increases as in a previous study (Song et al., 2021). The G-PE-induced unintended substitutions slightly increased only at the *RUNX1* site (Supplementary Figure S7A), but there were far fewer fold increases in these unintended edits than in the intended edits (Figure 1H). Considering that PE-mediated editing relies on the Cas9 nickase and the indels generated by PE are observed in a previous study (Anzalone et al., 2019), we then detected the potential indels induced by the Cas9 nickase by analyzing the same 15 target sites and adopted adjusted fold increases too (Supplementary Figure S7B). The results showed that G-PE induced higher indel frequency compared with original PE, but the increased percentage of indels was comparable to that of intended edits (Figure 1H).

The off-target effect is another key issue for genome targeting. Therefore, we analyzed potential off-target sites of pegRNA and paired sgRNA of the PE3 system. We identified three endogenous sites and predicted potential off-target sites with 2–4 nucleotide mismatches individually for pegRNA and paired sgRNA by Cas-OFFinder (Supplementary Figure S8A, C, and E). The analysis showed that almost all off-target sites had <0.1% off-target editing (Supplementary Figure S8B, D, and F) and no statistical difference in the off-target effects between G-PE and the original PE was observed (Figure 1H). Therefore, G-PE can achieve more efficient editing while retaining its high fidelity without potential off-target effects.

Taken together, by systematically comparing the effects of appending different structural RNA motifs at the 3′ end of pegRNA, we have observed that G-quadruplex modification can effectively improve the efficiency of PE-mediated genome editing. Additionally, our studies established G-PE as a more efficient prime editing system and identified that G-PE broadly improves PE efficiency in all three cell lines, providing a new tool for genome editing. During the preparation of this paper, a recent study showed that incorporation of certain structured RNA motifs, including evopreQ_1_ and mpknot, G-quadruplexes, 15-bp hairpins, xrRNA, and the P4–P6 domain of the group I intron to the 3′ end of pegRNAs, significantly improves prime editing efficiencies (Nelson et al., 2022), confirming the reliability and usefulness of our study. Nevertheless, further extensive development is highly required.

*[Targeted amplicon sequencing data has been deposited in the NCBI under BioProject number SUB10748207. We thank members of the Huang lab for helpful discussions and thank the Molecular and Cell Biology Core Facility (MCBCF) at the School of Life Science and Technology, ShanghaiTech University, for providing technical support. This work was supported by the Emergency Key Program of Guangzhou Laboratory (EKPG21-18) and the Leading Talents of Guangdong Province Program (608285568031). X.H. conceived, designed, and supervised the project. X.L., X.W., and W.S. performed most experiments with the help of M.Z. and S.H. X.H. provided expert technical assistance. X.L. and X.H. wrote the paper with inputs from all the authors. X.H. managed the project.]*

## Supplementary Material

mjac022_Supplemental_File
