# Supplementary material for: Enhancing prime editing efficiency by modified pegRNA with RNA G-quadruplexes
Source: J Mol Cell Biol. 2022 Apr 12;14(4):mjac022. doi: 10.1093/jmcb/mjac022 (PMC9345309; doi:10.1093/jmcb/mjac022)
Supplement: mjac022_Supplemental_File [file mjac022_supplemental_file.pdf]

Supplementary material

Enhancing prime editing efficiency by modified pegRNA with RNA G-quadruplexes

Xiangyang Li[1,2,†], Xin Wang[1,2,†], Wenjun Sun[1,2,†], Shisheng Huang[1,2], Mingtian Zhong[3], Yuan Yao[4], Quanjiang Ji[5], and Xingxu Huang[1,6,*]

[1]Gene Editing Center, School of Life Science and Technology, ShanghaiTech University, 100 Haike Rd., Pudong New Area, Shanghai 201210, China
[2]University of Chinese Academy of Sciences, Beijing 100049, China
[3]Institute for Brain Research and Rehabilitation, Guangdong Key Laboratory of Mental Health and Cognitive Science, Center for Studies of Psychological Application, South China Normal University, Guangzhou 510631, China
[4]Hangzhou Global Scientific and Technological Innovation Center, Zhejiang University, Hangzhou 311215, China
[5]School of Physical Science and Technology, ShanghaiTech University, 100 Haike Rd., Pudong New Area, Shanghai 201210, China
[6]Guangzhou Laboratory, No. 9 XingDaoHuanBei Road, Guangzhou International Bio Island, Guangzhou 510005, China
[†]These authors contributed equally to this work.
*Correspondence to: Xingxu Huang, E-mail: huangxx@shanghaitech.edu.cn

Supplementary Materials and methods

Cell culture and transfection

HEK293T, HeLa, and U2OS cell lines were cultured in Dulbecco's Modified Eagle's Medium supplemented with 10% (v/v) fetal bovine serum (FBS) at 37 °C with 5% CO2. For plasmid transfection, cells were seeded in 24-well a day before and transfected at about 70% density. A total of 900 ng PE2 plasmids, 300 ng pegRNA plasmids and 100 ng corresponding nick sgRNA plasmids were transfected into cells with EZ Trans (Shanghai Life iLab Biotech Co., Ltd) or Lipofectamine 2000 (Life Technologies) according to manufactures' instruction. 3 days after transfection, cells were harvested from Fluorescence Activating Cell Sorter (FACS).

Plasmid construction

Prime editing system plasmid was purchased from Addgene (Addgene, #132775). pegRNA plasmid was constructed according to the methods described in our previous study (Liu et al., 2020). To construct modified pegRNA plasmids, the plasmid backbone was amplified from pGL3-U6-sgRNA-EGFP (Addgene, #107721) using Phanta® Max Super Fidelity DNA Polymerase (Vazyme) (Supplementary Table S1). The backbone amplicon was then cut by BsaI-HFv2 (NEB) for overhangs. Spacer oligos (the top strand oligo includes 5' ACCG and 3' GTTTT overhangs, while the bottom strand oligo comprises a 5' CTCTAAAAC overhang), pegRNA 3' extension with RNA structural motifs (the top strand oligo included 5' GTGC overhang while the bottom strand oligo included 5' AAAC overhang), the spacer of nick-sgRNA (Supplementary Table S2), and sgRNA scaffold oligos (featuring compatible overhangs) were synthesized (Supplementary Table S1).

Flow cytometry

Cells were harvested for flow cytometry 3 days after transfection. pegRNAs were cloned into pGL3-U6-sgRNA-EGFP, and GFP signal was detected with flow cytometry. Similarly, sgRNAs were annealed into pGL3-U6-sgRNA-mCherry, and mCherry signal was detected with flow cytometry. Prime editor, together with pegRNA and sgRNA plasmids were transfected simultaneously. Total 10,000 GFP-mCherry double positive cells were collected by FACS for genomic genotyping.

Genomic DNA extraction and genotyping

The genomic DNA of GFP-mCherry+ cells was extracted using QuickExtract™ DNA Extraction Solution (Lucigen) according to manufacturer's protocols. The isolated DNA was PCR-amplified with Phanta® Max Super-Fidelity DNA Polymerase (Vazyme). Primers used are listed in (Supplementary Table S3).

Targeted deep-sequencing

Target sites were amplified with Phanta® Max Super-Fidelity DNA Polymerase (Vazyme) and subjected into high-throughput sequencing (2×150, paired-end) with Illumina Hiseq X Ten platform.

The amplicons were analyzed with CRISPResso2. The primers used for editing efficiency determination are listed in (Supplementary Table S3).

Off-target analysis

Potential off-target sites were predicted in human genome (GRCh38/hg38) with Cas-OFFinder tools (http://www.rgenome.net/cas-offinder), and the region around off-target sites were amplified with Phanta® Max Super-Fidelity DNA Polymerase (Vazyme), and subjected into high-throughput sequencing with Illumina Hiseq X Ten platform. The amplicons were analyzed with CRIPResso2 and the off-target sites are listed in (Supplementary Figure 8A, C and E). Primers used are listed in (Supplementary Table S4).

Analysis of adjusted fold increase

Adjusted fold increase was calculated according to the methods described in previous study (Song et al., 2021). In some cases, we calculated an adjusted fold increase in which +0.1% was added to both the G-PE and original PE efficiencies in order to avoid mathematical errors and attenuate insignificant fold increases as shown below.

$$\text{Adjusted fold increase} = \frac{\text{G} - \text{PE efficiency } (\%) + 0.1\ \%}{\text{Original PE } (\%) + 0.1\ \%}$$

When we used an adjusted fold increase, we mention this point in the legends to the relevant figures.

Data analysis

All data were calculated based on three independent experiments, and Students' *t*-test (two-tailed) were used to calculated statistical difference. Mean was showed values ± SD.

Data availability

Targeted amplicon sequencing data has been deposited in the NCBI under BioProject number SUB10748207

Supplementary References

Liu, Y., Li, X., He, S., et al. (2020). Efficient generation of mouse models with the prime editing system. Cell Discov. *6*, 27.

Song, M., Lim, J.M., Min, S., et al. (2021). Generation of a more efficient prime editor 2 by addition of the Rad51 DNA-binding domain. Nat. Commun. *12*, 5617.

Supplementary Figures

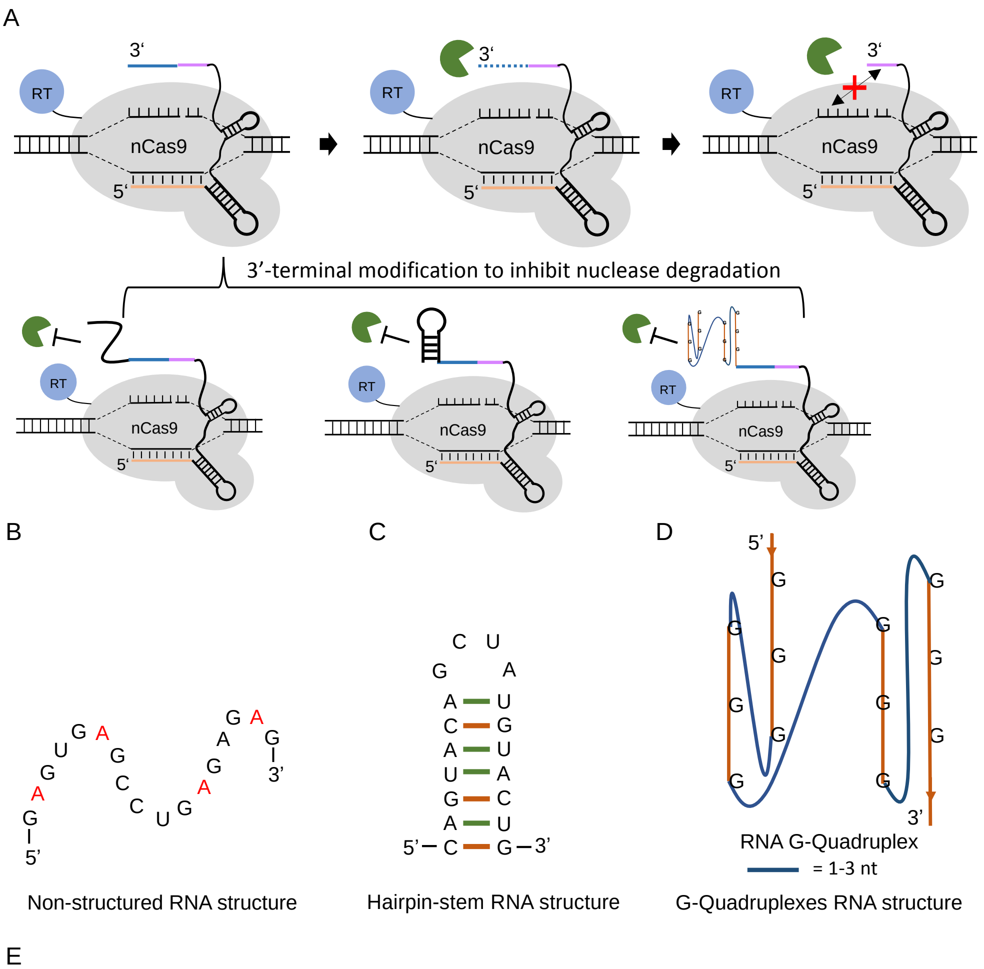

Origin	Sequence	Type	*Tm* (℃)
Non-structured	GAGUGAGCCUGAGAGAG	Non-structured RNA	52
Hairpin-stem	CAGUACAGAUCUGUACUG	Hairpin-stem RNA	48
Human telomerase RNA	GGGUGGGCCUGGGAGGG	G-quadruplex RNA	64
MT3	GGGAGGGAGGGAGAGGG	G-quadruplex RNA	71
TERRA	GGGUUAGGGUUAGGGUUAGGG	G-quadruplex RNA	68
NRAS	GGGAGGGGCGGGUCUGGG	G-quadruplex RNA	58

Supplementary Figure S1. The optimization of pegRNA with RNA structural motifs.

A. Up, a schematic of a PE complex and degradation of the 3′ extension of a pegRNA by exonucleases could impede the combination of unwinding ssDNA and 3′extension RNA. Down, a schematic of the pegRNA with three types of designed 3' terminal modification to inhibit nuclease degradation. Green graph represents exonucleases and an original pegRNA consists of spacer (orange), scaffold (black), RT (blue), and PBS (purple).

B. Non-structured RNA structure. Red nucleotides represent mutated base position compared with hTR G-quadruplex sequence.

C. Hairpin-stem RNA structure.

D. G-quadruplexes RNA structure. Blue line respect 1-3 nt base.

E. Characteristic of structured RNA motifs.

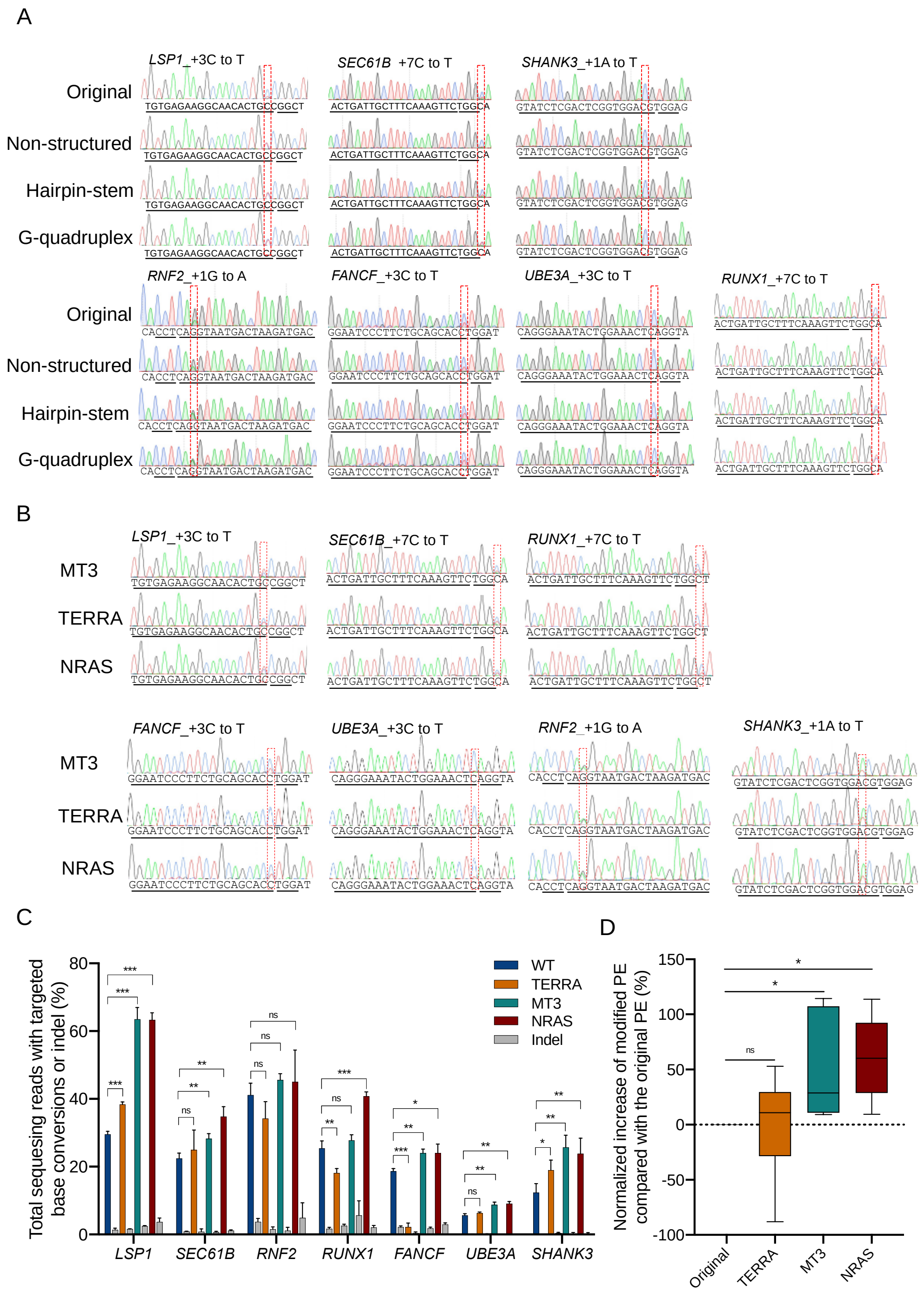

Supplementary Figure S2. Modified pegRNA with different genomic G-quadruplex also enhances prime editing activity in endogenous sites.

A. Sanger sequencing of the editing efficiency of endogenous sites with modified pegRNA in HEK293T cell. The frame in red indicated the targeted base.

B. Sanger sequencing of the editing of endogenous sites with *MT3*, *TERRA*, and *NRAS* G-quadruplex modified pegRNAs in HEK293T cell.

C. *MT3*, *TERRA*, and *NRAS* G-quadruplex modified pegRNA observably enhances prime editing activity in major endogenous sites.

D. Prime editing efficiency of modified pegRNA normalized to the increased efficiency of original pegRNA in **A**. Mean values ± SD, n = 3 independent experiments. (*$P < 0.05$, **$P < 0.005$, ***$P < 0.0005$)

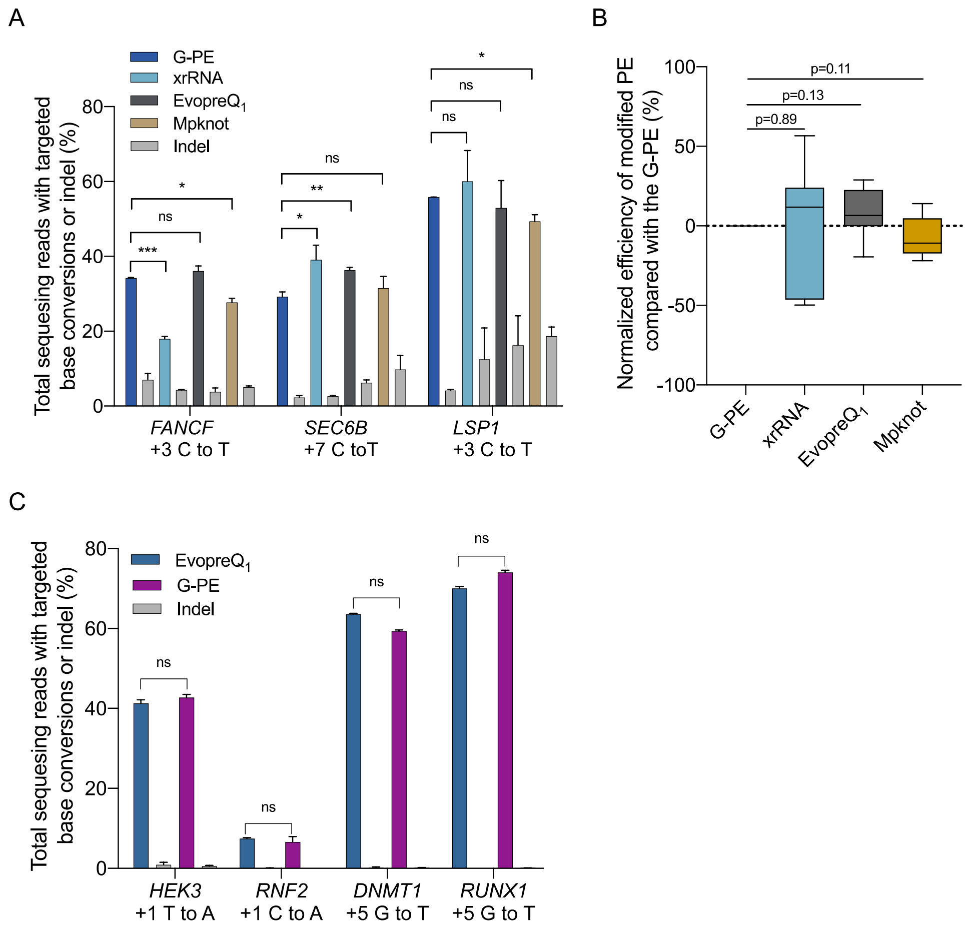

Supplementary Figure S3. Editing efficiencies of G-PE comparing with other modifications in HEK293T cells.

A. Analysis of G-PE editing efficiency comparing with evopreQ$_1$, mpknot and xrRNA at three endogenous sites.

B. Prime editing efficiency of other modifications normalized to the increased efficiency of G-PE.

C. Analysis of G-PE editing efficiency comparing with evopreQ$_1$ at four reported sites.

Mean values ± SD, n = 3 independent experiments. (*$P < 0.05$, **$P < 0.005$, ***$P < 0.0005$)

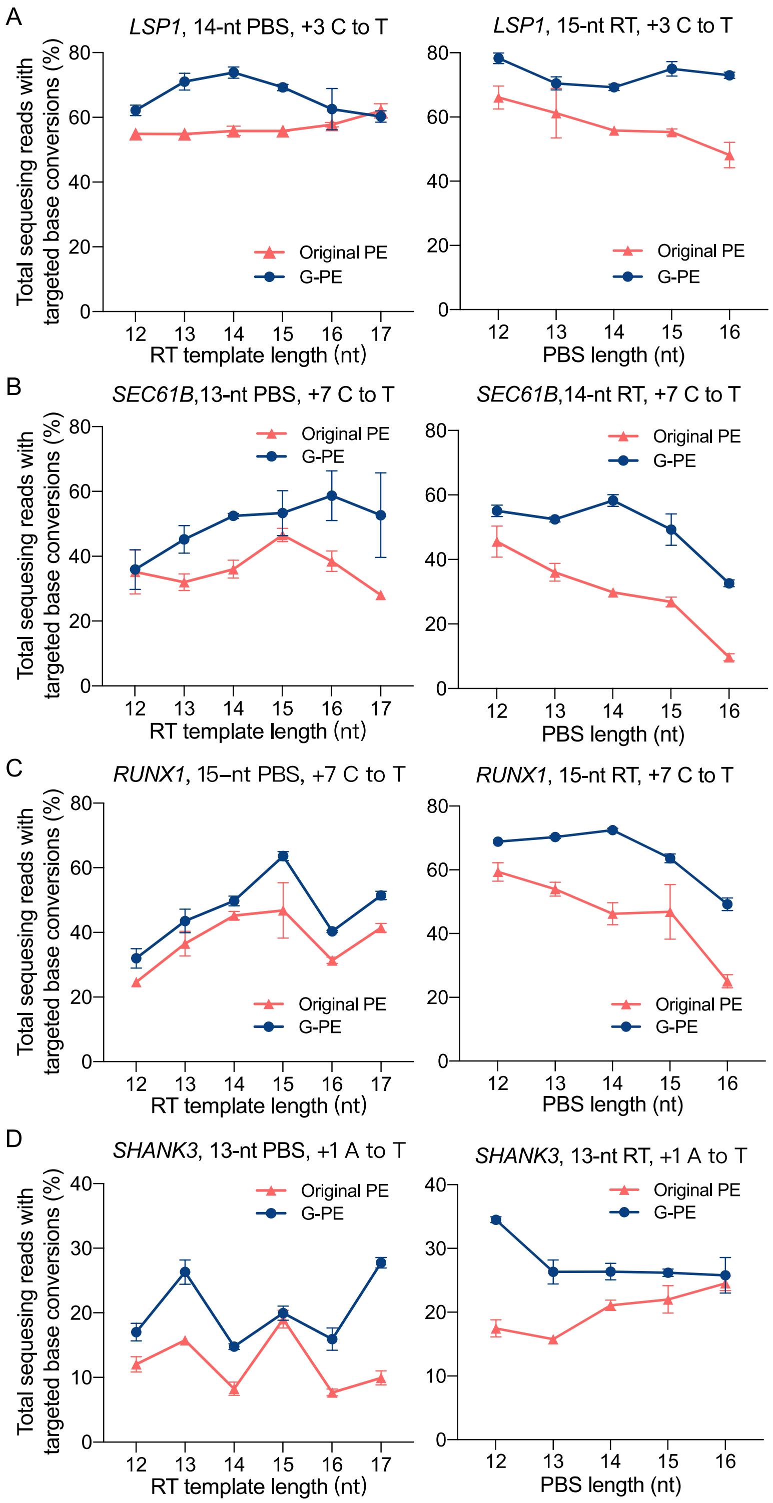

Supplementary Figure S4. G-PE editing efficiencies with various PBS and RT template lengths at four endogenous sites in HEK293T cells.

A–D. G-PE editing efficiencies with various PBS and RT template lengths comparing with original PE in *LSP1* (A), *SEC61B* (B), *RUNX1* (C), and *SHANK3* (D) sites.

Mean values ± SD, n = 3 independent experiments.

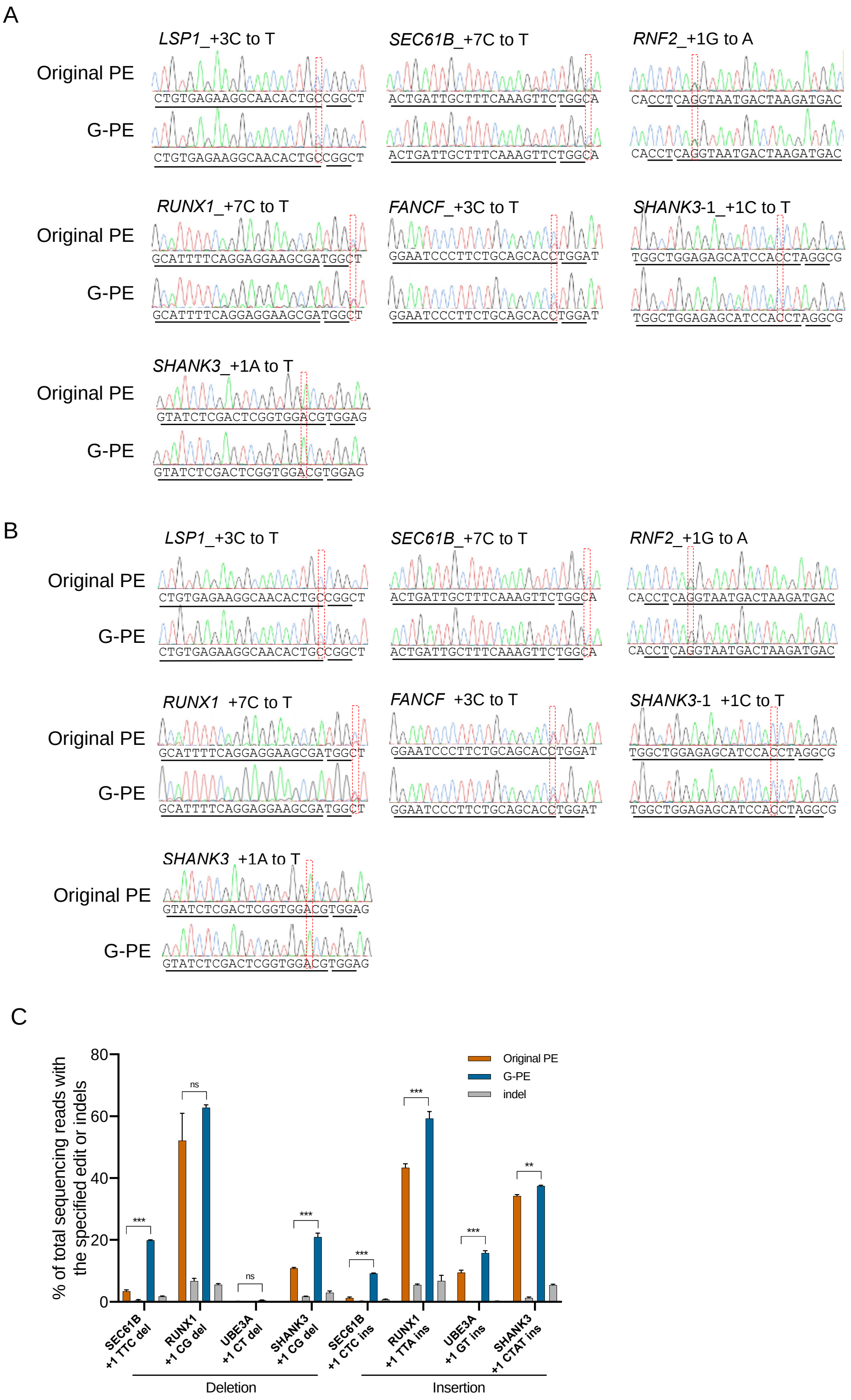

Supplementary Figure S5. Increasing targeted base substitutions, deletion and insertion efficiency by prime editing with G-PE in HeLa and U2OS cells.

A and B. Sanger sequencing chromatograms of targeted base conversions with original PE and G-PE of 7 sites in human HeLa (A) and U2OS (B) cell line. The frame in red indicated the targeted base.

C. The comparison of deletion and insertion efficiency of G-PE and original PE at 4 endogenous sites in human HeLa cell lines.

Mean values ± SD, n = 3 independent experiments. (*$P < 0.05$, **$P < 0.005$, ***$P < 0.0005$)

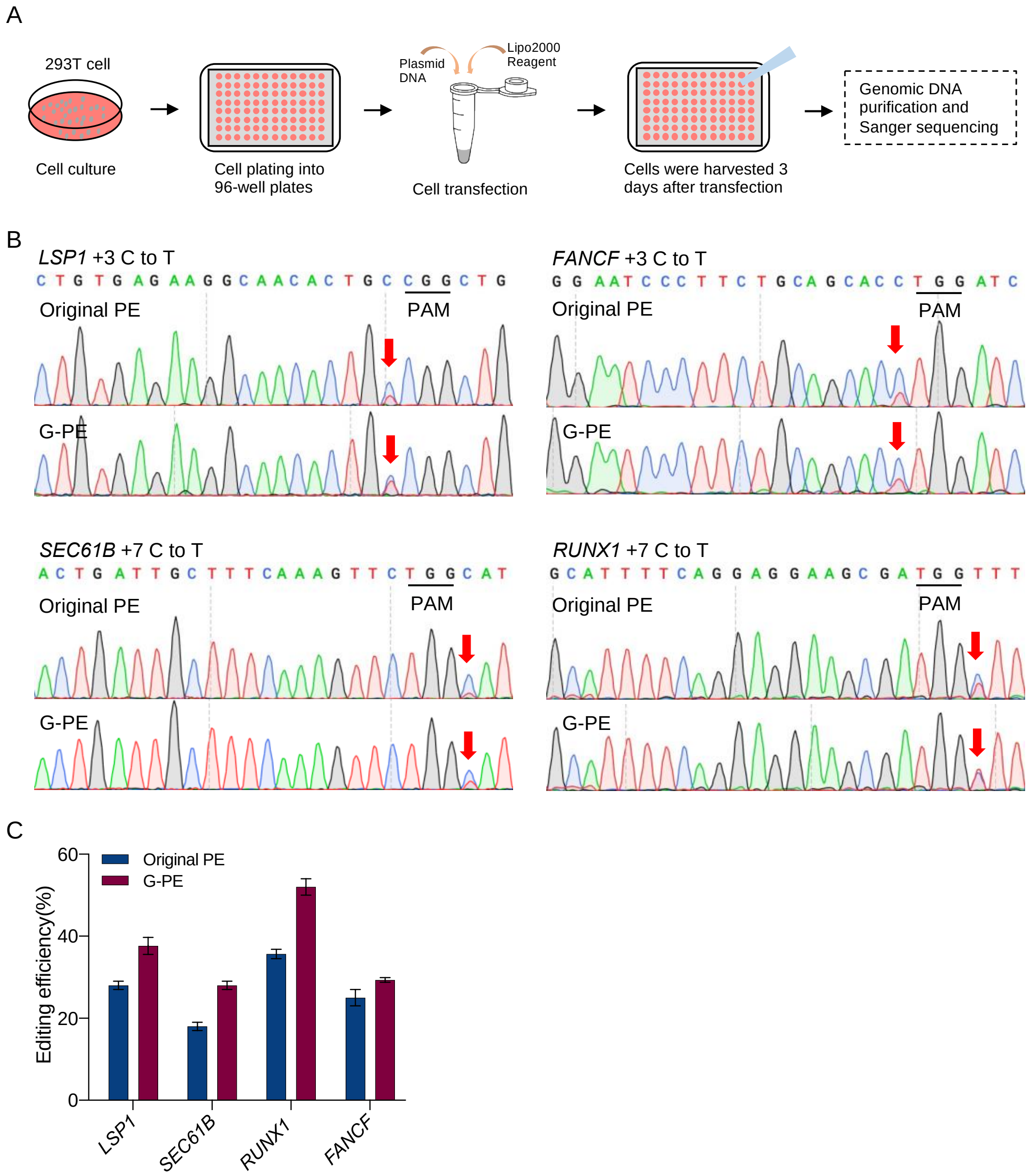

Supplementary Figure S6. G-PE editing efficiencies without sorting at four endogenous sites in HEK293T cells.

A. Schematic of our experiment steps to show G-PE editing efficiencies without sorting.

B. Sanger sequencing chromatograms of targeted base conversions with original PE and G-PE without sorting at four endogenous sites. The arrow in red indicated the targeted base.

C. The comparison of efficiency of G-PE and original PE at four endogenous sites without sorting. Mean values ± SD, n = 3 independent experiments.

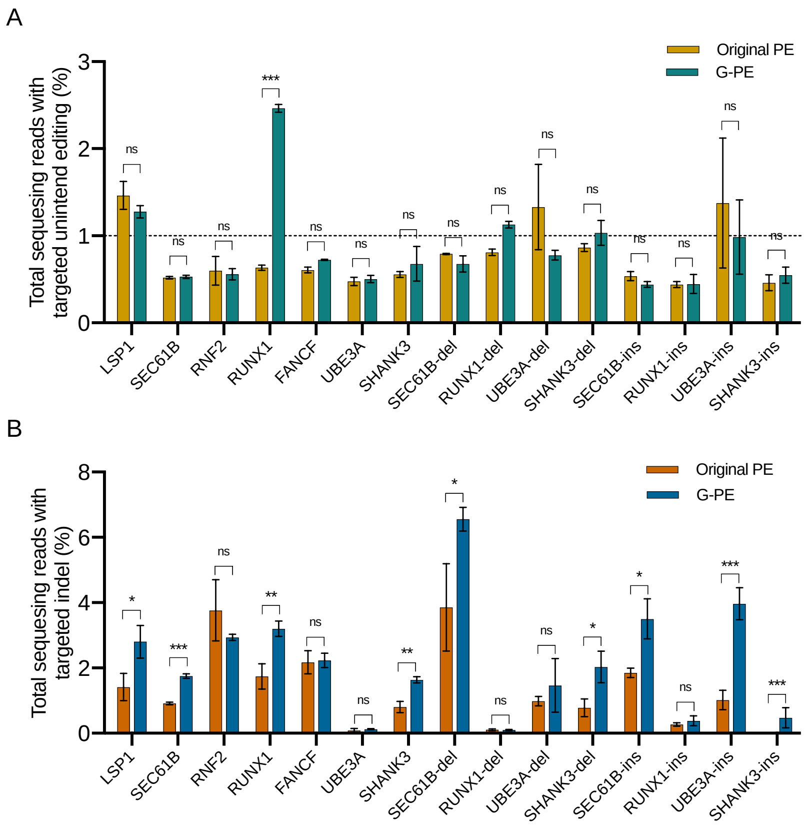

Supplementary Figure S7. Analysis of unintended edits and indels induced by prime editing with G-PE and original PE.

A. Analysis of unintended editing induced by prime editing with G-PE and original PE.

B. Analysis of indels induced by prime editing with G-PE and original PE.

Mean values ± SD, n = 3 independent experiments. (*$P < 0.05$, **$P < 0.005$, ***$P < 0.0005$)

A

No.	sgRNA sequence	Chr.	Start position	End position	Mismatches
SEC61B-pOT1	ACTGtTTGCTTTaAAtGTTC	chr15	91540267	91540267	3
SEC61B-pOT2	ACTGATTatTTTCAAAtTTC	chr20	13120456	13120456	3
SEC61B-pOT3	AgTGAaTGCTTTcAAAGTTC	chr1	77835366	77835366	3
SEC61B-pOT4	tCTaATTGCTTTCAgAGTTC	chr2	85262708	85262708	3
SEC61B-pOT5	ACTGATTGCTgTcAAAgTTC	chr16	4727805	4727805	3
SEC61B-nOT1	GCaCTGCTggATGACGGAGt	chr5	176270613	176270613	4
SEC61B-nOT2	GCCgTagTGCATGACGGtGT	chr17	78124147	78124147	4
SEC61B-nOT3	TCCCTGCTtCcTcAtGGAGT	chr9	35399333	35399333	4
SEC61B-nOT4	GagtTGCTACATGAaGGAGT	chr11	89421041	89421041	4
SEC61B-nOT5	GaCCTGCTaCATGAcGaAGT	chr4	109556768	109556768	4
SEC61B-nOT6	aaCCTGCTACAaGACGGAGt	chr22	17998973	17998973	4

B

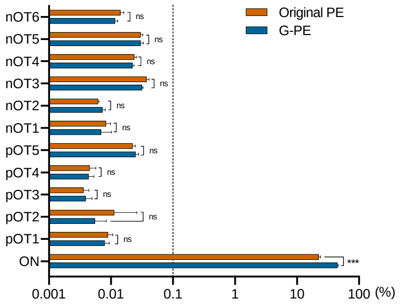

Off-target analysis of *SEC61B* of prime editing

C

No.	sgRNA sequence	Chr.	Start position	End position	Mismatches
RNF2-pOT1	GTCATCcTAGTgcTTACCTG	chr8	755960	755960	3
RNF2-pOT2	GTCATCtTAGTCATTtaCTG	chr17	17168758	17168758	3
RNF2-pOT3	GTaATaTTAGTCATTACCgG	chr6	142890925	142890925	3
RNF2-pOT4	GTCATCTgAGgCATTAcCTG	chr6	150919928	150919928	3
RNF2-pOT5	GTaATCTgAGTCATTaCCTG	chr10	129047187	129047187	3
RNF2-nOT1	TCAACCATTtAGCAAAAacT	chr13	71847894	71847894	3
RNF2-nOT2	cCAACCATTcAGCAAtACAc	chr20	3519195	3519195	3
RNF2-nOT3	TcAACCATTgAGCAAgAaAT	chr1	22721327	22721327	3
RNF2-nOT4	TgAAaCCTTAAGCAAAACAt	chr22	29440921	29440921	3

D

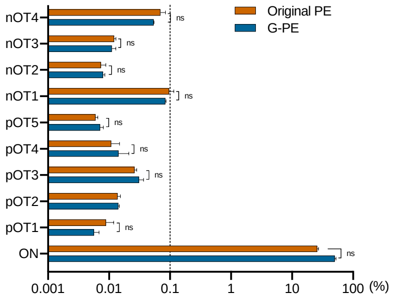

Off-target analysis of *RNF2* of prime editing

E

No.	sgRNA sequence	Chr.	Start position	End position	Mismatches
RUNX1-pOT1	GCATTTTtAGaAGGAAGgGA	chr1	158024825	158024825	3
RUNX1-pOT2	GCATTTTtAGGAaGAAGtGA	chr4	140191403	140191403	3
RUNX1-pOT3	GtcTTTTCAGGAGGAAGtGA	chr11	111027018	111027018	3
RUNX1-pOT4	GCATTTTgAGGAGaAAGCgA	chr3	120258754	120258754	3
RUNX1-pOT5	GCATTTTCAGGAGGAAtCcA	chr1	29436495	29436495	2
RUNX1-nOT1	cTGAAGCACTGTGGGgAcGA	chr5	65031327	65031327	3
RUNX1-nOT2	ATGAAGCACTtTGGGTAgcA	chr10	56087088	56087088	3
RUNX1-nOT3	ATGtAGaACTGTGGGTACcA	chr8	2140089	2140089	3
RUNX1-nOT4	AaGAAGCAaTGTGGGTAcGa	chr12	30978679	30978679	4
RUNX1-nOT5	ATGcAGCcCTGTGGtTcCGA	chr21	46539761	46539761	4

F

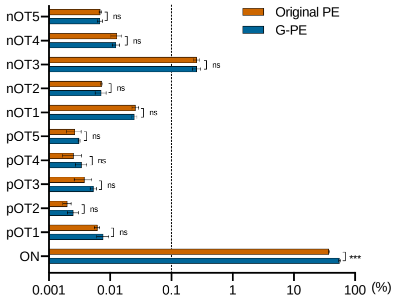

Off-target analysis of *RUNX1* of prime editing

Supplementary Figure S8. Off-target analysis of prime editing induced by G-PE and original PE in three endogenous sites.

A, B. Information and off-target analysis of *SEC61B*.

C, D. Information and off-target analysis of *RNF2*.

E, F. Information and off-target analysis of *RUNX1*.

The letter in lower case indicates the mismatched sequence. Mean values ± SD, n = 3 independent experiments. (**P* < 0.05, ***P* < 0.005, ****P* < 0.0005)

Supplementary Tables

Supplementary Table S1. Primers used for constructing of G-quadruplex modified pegRNA plasmids.

backbone_pegRNA-F	AGCTAGGTCTCCTTTTTTTAAAGAATTCTCGACCTCGAGAC
backbone_pegRNA-R	TCTCTCGGTCTCACGGTGTTTCGT
sg_scaffold-top	AGAGCTAGAAATAGCAAGTTAAAATAAGGCTAGTCCGTTATCAACTTGAAAAAGTGGCACCGAGTCG
sg_scaffold-bottom	GCACCGACTCGGTGCCACTTTTTCAAGTTGATAACGGACTAGCCTTATTTTAACTTGCTATTTCTAG

Supplementary Table S2. pegRNA target sites, RT template, PBS sequences, modification.

pegRNA	pegRNA target sequence	Sequence for RT template, PBS and modification	Nick sg target sequence	Intended edit
LSP1	CTGTGAGAAGGCAACACTGC**CGG**	CAGCCCCAGCCGACAGTGTTGCCTTCTCA	CCTCTCTGGGCCCTTTGGGT	+3 C to T
SEC61B-T1	ACTGATTGCTTTCAAAGTT**CTGG**	GTGTTCATACCAGAACTTTGAAAGCAAT	GCCCTGCTACATGACGGAGT	+7 C to T
RNF2	**CCT**CAGGTAATGACTAAGATGAC	AACGAACACCTCAAGTAATGACTAAGATG	TCAACCATTAAGCAAAACAT	+1 G to A
RUNX1-T1	GCATTTTCAGGAGGAAGCGA**TGG**	TGTCTGAAACCATCGCTTCCTCCTGAAAAT	ATGAAGCACTGTGGGTACGA	+7 C to T
FANCF	GGAATCCCTTCTGCAGCACC**TGG**	AAAAGCGATCCAAGTGCTGCAGAAGGGA	GGGGTCCCAGGTGCTGACGT	+3 C to T
UBE3A-T1	CAGGGAAATACTGGAAACTC**AGG**	ATGAGTACCTAAGTTTCCAGTATTTC	ACCACTAATCCCCAGATTTT	+3 C to T
SHANK3	GTATCTCGAGTCGGTGGACG**TGG**	ACACCCTCCACGACCACCGACTCGAG	AGAACGACCTCACCTCGATG	+1 A to T
LSP1-NS	CTGTGAGAAGGCAACACTGC**CGG**	CAGCCCCAGCCGACAGTGTTGCCTTCTCAGAGTGAGCCTGAGAGAG	CCTCTCTGGGCCCTTTGGGT	+3 C to T
SEC61B-T1-NS	ACTGATTGCTTTCAAAGTT**CTGG**	GTGTTCATACCAGAACTTTGAAAGCAATGAGTGAGCCTGAGAGAG	GCCCTGCTACATGACGGAGT	+7 C to T
RNF2-NS	**CCT**CAGGTAATGACTAAGATGAC	AACGAACACCTCAAGTAATGACTAAGATGGAGTGAGCCTGAGAGAG	TCAACCATTAAGCAAAACAT	+1 G to A
RUNX1-T1-NS	GCATTTTCAGGAGGAAGCGA**TGG**	TGTCTGAAACCATCGCTTCCTCCTGAAAATGAGTGAGCCTGAGAGAG	ATGAAGCACTGTGGGTACGA	+7 C to T
FANCF-NS	GGAATCCCTTCTGCAGCACC**TGG**	AAAAGCGATCCAAGTGCTGCAGAAGGGAGAGTGAGCCTGAGAGAG	GGGGTCCCAGGTGCTGACGT	+3 C to T
UBE3A-T1-NS	CAGGGAAATACTGGAAACTC**AGG**	ATGAGTACCTAAGTTTCCAGTATTTCGAGTGAGCCTGAGAGAG	ACCACTAATCCCCAGATTTT	+3 C to T
SHANK3-NS	GTATCTCGAGTCGGTGGACG**TGG**	ACACCCTCCACGACCACCGACTCGAGGAGTGAGCCTGAGAGAG	AGAACGACCTCACCTCGATG	+1 A to T
LSP1-HS	CTGTGAGAAGGCAACACTGC**CGG**	CAGCCCCAGCCGACAGTGTTGCCTTCTCACAGTACAGAUCTGTACTG	CCTCTCTGGGCCCTTTGGGT	+3 C to T
SEC61B-T1-HS	ACTGATTGCTTTCAAAGTT**CTGG**	GTGTTCATACCAGAACTTTGAAAGCAATCAGTACAGAUCTGTACTG	GCCCTGCTACATGACGGAGT	+7 C to T
RNF2-HS	**CCT**CAGGTAATGACTAAGATGAC	AACGAACACCTCAAGTAATGACTAAGATGCAGTACAGAUCTGTACTG	TCAACCATTAAGCAAAACAT	+1 G to A
RUNX1-T1-HS	GCATTTTCAGGAGGAAGCGA**TGG**	TGTCTGAAACCATCGCTTCCTCCTGAAAATCAGTACAGAUCTGTACTG	ATGAAGCACTGTGGGTACGA	+7 C to T
FANCF-HS	GGAATCCCTTCTGCAGCACC**TGG**	AAAAGCGATCCAAGTGCTGCAGAAGGGACAGTACAGAUCTGTACTG	GGGGTCCCAGGTGCTGACGT	+3 C to T
UBE3A-T1-HS	CAGGGAAATACTGGAAACTC**AGG**	ATGAGTACCTAAGTTTCCAGTATTTCCAGTACAGAUCTGTACTG	ACCACTAATCCCCAGATTTT	+3 C to T
SHANK3-HS	GTATCTCGAGTCGGTGGACG**TGG**	ACACCCTCCACGACCACCGACTCGAGCAGTACAGAUCTGTACTG	AGAACGACCTCACCTCGATG	+1 A to T

LSP1-hTR	CTGTGAGAAGGCAACACT GC**CGG**	CAGCCCCAGCCGACAGTGTTGCCTTCTCAGG GTGGGCCTGGGAGGG	CCTCTCTGGGCCC TTTGGGT	+3 C to T
SEC61B-T1-hTR	ACTGATTGCTTTCAAAGTT C**TGG**	GTGTTCATACCAGAACTTTGAAAGCAATGGG TGGGCCTGGGAGGG	GCCCTGCTACATG ACGGAGT	+7 C to T
RNF2-hTR	**CCT**CAGGTAATGACTAAG ATGAC	AACGAACACCTCAAGTAATGACTAAGATGGG GTGGGCCTGGGAGGG	TCAACCATTAAGCA AAACAT	+1 G to A
RUNX1-T1-hTR	GCATTTTCAGGAGGAAGC GA**TGG**	TGTCTGAAACCATCGCTTCCTCCTGAAAATG GGTGGGCCTGGGAGGG	ATGAAGCACTGTG GGTACGA	+7 C to T
FANCF-hTR	GGAATCCCTTCTGCAGCA CC**TGG**	AAAAGCGATCCAAGTGCTGCAGAAGGGAGG GTGGGCCTGGGAGGG	GGGGTCCCAGGTG CTGACGT	+3 C to T
UBE3A -hTR	CAGGGAAATACTGGAAAC TC**AGG**	ATGAGTACCTAAGTTTCCAGTATTTCGGGTG GGCCTGGGAGGG	ACCACTAATCCCC AGATTTT	+3 C to T
SHANK3-hTR	GTATCTCGAGTCGGTGGA CG**TGG**	ACACCCTCCACGACCACCGACTCGAGGGGT GGGCCTGGGAGGG	AGAACGACCTCAC CTCGATG	+1 A to T
LSP1-MT3	CTGTGAGAAGGCAACACT GC**CGG**	CAGCCCCAGCCGACAGTGTTGCCTTCTCAGG GAGGGAGGGAGAGGG	CCTCTCTGGGCCC TTTGGGT	+3 C to T
SEC61B-T1-MT3	ACTGATTGCTTTCAAAGTT C**TGG**	GTGTTCATACCAGAACTTTGAAAGCAATGGG AGGGAGGGAGAGGG	GCCCTGCTACATG ACGGAGT	+7 C to T
RNF2-MT3	**CCT**CAGGTAATGACTAAG ATGAC	AACGAACACCTCAAGTAATGACTAAGATGGG GAGGGAGGGAGAGGG	TCAACCATTAAGCA AAACAT	+1 G to A
RUNX1-T1-MT3	GCATTTTCAGGAGGAAGC GA**TGG**	TGTCTGAAACCATCGCTTCCTCCTGAAAATG GGAGGGAGGGAGAGGG	ATGAAGCACTGTG GGTACGA	+7 C to T
FANCF-MT3	GGAATCCCTTCTGCAGCA CC**TGG**	AAAAGCGATCCAAGTGCTGCAGAAGGGAGG GAGGGAGGGAGAGGG	GGGGTCCCAGGTG CTGACGT	+3 C to T
UBE3A-T1-MT3	CAGGGAAATACTGGAAAC TC**AGG**	ATGAGTACCTAAGTTTCCAGTATTTCGGGAG GGAGGGAGAGGG	ACCACTAATCCCC AGATTTT	+3 C to T
SHANK3 -MT3	GTATCTCGAGTCGGTGGA CG**TGG**	ACACCCTCCACGACCACCGACTCGAGGGGA GGGAGGGAGAGGG	AGAACGACCTCAC CTCGATG	+1 A to T
LSP1-TERRA	CTGTGAGAAGGCAACACT GC**CGG**	CAGCCCCAGCCGACAGTGTTGCCTTCTCAGG GTTAGGGTTAGGGTTAGGG	CCTCTCTGGGCCC TTTGGGT	+3 C to T
SEC61B-T1-TERRA	ACTGATTGCTTTCAAAGTT C**TGG**	GTGTTCATACCAGAACTTTGAAAGCAATGGG TTAGGGTTAGGGTTAGGG	GCCCTGCTACATG ACGGAGT	+7 C to T
RNF2-TERRA	**CCT**CAGGTAATGACTAAG ATGAC	AACGAACACCTCAAGTAATGACTAAGATGGG GTTAGGGTTAGGGTTAGGG	TCAACCATTAAGCA AAACAT	+1 G to A
RUNX1-T1-TERRA	GCATTTTCAGGAGGAAGC GA**TGG**	TGTCTGAAACCATCGCTTCCTCCTGAAAATG GGTTAGGGTTAGGGTTAGGG	ATGAAGCACTGTG GGTACGA	+7 C to T
FANCF-TERRA	GGAATCCCTTCTGCAGCA CC**TGG**	AAAAGCGATCCAAGTGCTGCAGAAGGGAGG GTTAGGGTTAGGGTTAGGG	GGGGTCCCAGGTG CTGACGT	+3 C to T
UBE3A-T1-TERRA	CAGGGAAATACTGGAAAC TC**AGG**	ATGAGTACCTAAGTTTCCAGTATTTCGGGTTA GGGTTAGGGTTAGGG	ACCACTAATCCCC AGATTTT	+3 C to T
SHANK3-TERRA	GTATCTCGAGTCGGTGGA CG**TGG**	ACACCCTCCACGACCACCGACTCGAGGGGT TAGGGTTAGGGTTAGGG	AGAACGACCTCAC CTCGATG	+1 A to T
LSP1-NRAS	CTGTGAGAAGGCAACACT GC**CGG**	CAGCCCCAGCCGACAGTGTTGCCTTCTCAGG GAGGGGCGGGTCTGGG	CCTCTCTGGGCCC TTTGGGT	+3 C to T
SEC61B-T1-NRAS	ACTGATTGCTTTCAAAGTT C**TGG**	GTGTTCATACCAGAACTTTGAAAGCAATGGG AGGGGCGGGTCTGGG	GCCCTGCTACATG ACGGAGT	+7 C to T
RNF2-NRAS	**CCT**CAGGTAATGACTAAG ATGAC	AACGAACACCTCAAGTAATGACTAAGATGGG GAGGGGCGGGTCTGGG	TCAACCATTAAGCA AAACAT	+1 G to A
RUNX1-T1-NRAS	GCATTTTCAGGAGGAAGC GA**TGG**	TGTCTGAAACCATCGCTTCCTCCTGAAAATG GGAGGGGCGGGTCTGGG	ATGAAGCACTGTG GGTACGA	+7 C to T
FANCF-NRAS	GGAATCCCTTCTGCAGCA CC**TGG**	AAAAGCGATCCAAGTGCTGCAGAAGGGAGG GAGGGGCGGGTCTGGG	GGGGTCCCAGGTG CTGACGT	+3 C to T
UBE3A-T1-NRAS	CAGGGAAATACTGGAAAC TC**AGG**	ATGAGTACCTAAGTTTCCAGTATTTCGGGAG GGGCGGGTCTGGG	ACCACTAATCCCC AGATTTT	+3 C to T
SHANK3 -NRAS	GTATCTCGAGTCGGTGGA CG**TGG**	ACACCCTCCACGACCACCGACTCGAGGGGA GGGGCGGGTCTGGG	AGAACGACCTCAC CTCGATG	+1 A to T
SEC61B-T2	ACTGATTGCTTTCAAAGTT C**TGG**	GTGTTCATGCCACTTTGAAAGCAAT	GCCCTGCTACATG ACGGAGT	+1 TTC del
RUNX1-T2	GCATTTTCAGGAGGAAGC GA**TGG**	TGTCTGAAGCCATCTTCCTCCTGAAAAT	ATGAAGCACTGTG GGTACGA	+1 CG del
UBE3A-T2	CAGGGAAATACTGGAAAC TC**AGG**	ATGAGTACCTGTTTCCAGTATTTC	ACCACTAATCCCC AGATTTT	+1 CT del
SHANK3-T2	GTATCTCGAGTCGGTGGA CG**TGG**	ACACCCTCCATCCACCGACTCGAG	AGAACGACCTCAC CTCGATG	+2 CG del
SEC61B-T3	ACTGATTGCTTTCAAAGTT C**TGG**	GTGTTCATGCCAGAACTTTGAAAGCAAT	GCCCTGCTACATG ACGGAGT	+1 CTC ins

RUNX1-T3	GCATTTTCAGGAGGAAGCGA**TGG**	TGTCTGAAGCCATCGTAACTTCCTCCTGAAAAT	ATGAAGCACTGTGGGTACGA	+1 TTA ins
UBE3A-T3	CAGGGAAATACTGGAAACTC**AGG**	ATGAGTACCTGAGGTTTTCCAGTATTTC	ACCACTAATCCCCAGATTTT	+1 GT ins
SHANK3-T3	GTATCTCGAGTCGGTGGACG**TGG**	ACACCCTCCACGTATAGCCACCGACTCGAG	AGAACGACCTCACCTCGATG	+1 CTAT ins
SEC61B-T2-hTR	ACTGATTGCTTTCAAAGTTC**TGG**	GTGTTCATGCCACTTTGAAAGCAATGGGTGGGCCTGGGAGGG	GCCCTGCTACATGACGGAGT	+1 TTC del
RUNX1-T2-hTR	GCATTTTCAGGAGGAAGCGA**TGG**	TGTCTGAAGCCATCTTCCTCCTGAAAATGGGTGGGCCTGGGAGGG	ATGAAGCACTGTGGGTACGA	+1 CG del
UBE3A-T2-hTR	CAGGGAAATACTGGAAACTC**AGG**	ATGAGTACCTGTTTCCAGTATTTCGGGTGGGCCTGGGAGGG	ACCACTAATCCCCAGATTTT	+1 CT del
SHANK3-T2-hTR	GTATCTCGAGTCGGTGGACG**TGG**	ACACCCTCCATCCACCGACTCGAGGGGTGGGCCTGGGAGGG	AGAACGACCTCACCTCGATG	+2 CG del
SEC61B-T3-hTR	ACTGATTGCTTTCAAAGTTC**TGG**	GTGTTCATGCCAGAACTTTGAAAGCAATGGGTGGGCCTGGGAGGG	GCCCTGCTACATGACGGAGT	+1 CTC ins
RUNX1-T3-hTR	GCATTTTCAGGAGGAAGCGA**TGG**	TGTCTGAAGCCATCGTAACTTCCTCCTGAAAATGGGTGGGCCTGGGAGGG	ATGAAGCACTGTGGGTACGA	+1 TTA ins
UBE3A-T3-hTR	CAGGGAAATACTGGAAACTC**AGG**	ATGAGTACCTGAGGTTTTCCAGTATTTCGGGTGGGCCTGGGAGGG	ACCACTAATCCCCAGATTTT	+1 GT ins
SHANK3-T3-hTR	GTATCTCGAGTCGGTGGACG**TGG**	ACACCCTCCACGTATAGCCACCGACTCGAGGGTGGGCCTGGGAGGG	AGAACGACCTCACCTCGATG	+1 CTAT ins
FANCF-xrRNA	GGAATCCCTTCTGCAGCACC**TGG**	AAAAGCGATCCAAGTGCTGCAGAAGGGATCTCTCTCGCGTAACCTCCATCCGAGTTGCAAGAGAGGGAAACGCAGTCTC	GGGGTCCCAGGTGCTGACGT	+3 C to T
FANCF-evopreQ1	GGAATCCCTTCTGCAGCACC**TGG**	AAAAGCGATCCAAGTGCTGCAGAAGGGATCTCTCTCTTGACGCGGTTCTATCTAGTTACGCGTTAAACCAACTAGAAA	GGGGTCCCAGGTGCTGACGT	+3 C to T
FANCF-mpknot	GGAATCCCTTCTGCAGCACC**TGG**	AAAAGCGATCCAAGTGCTGCAGAAGGGATCTCTCTCGGGTCAGGAGCCCCCCCCCTGAACCCAGGATAACCCTCAAAGTCGGGGGGCAACCC	GGGGTCCCAGGTGCTGACGT	+3 C to T
SEC61B-xrRNA	ACTGATTGCTTTCAAAGTTC**TGG**	GTGTTCATACCAGAACTTTGAAAGCAATTCTCTCTCGCGTAACCTCCATCCGAGTTGCAAGAGAGGGAAACGCAGTCTC	GCCCTGCTACATGACGGAGT	+7 C to T
SEC61B-evopreQ1	ACTGATTGCTTTCAAAGTTC**TGG**	GTGTTCATACCAGAACTTTGAAAGCAATTCTCTCTCTTGACGCGGTTCTATCTAGTTACGCGTTAAACCAACTAGAAA	GCCCTGCTACATGACGGAGT	+7 C to T
SEC61B-mpknot	ACTGATTGCTTTCAAAGTTC**TGG**	GTGTTCATACCAGAACTTTGAAAGCAATTCTCTCTCGGGTCAGGAGCCCCCCCCCTGAACCCAGGATAACCCTCAAAGTCGGGGGGCAACCC	GCCCTGCTACATGACGGAGT	+7 C to T
UBE3A-xrRNA	CAGGGAAATACTGGAAACTC**AGG**	ATGAGTACCTAAGTTTCCAGTATTTCTCTCTCTCGCGTAACCTCCATCCGAGTTGCAAGAGAGGGAAACGCAGTCTC	ACCACTAATCCCCAGATTTT	+3 C to T
UBE3A-evopreQ1	CAGGGAAATACTGGAAACTC**AGG**	ATGAGTACCTAAGTTTCCAGTATTTCTCTCTCTCTTGACGCGGTTCTATCTAGTTACGCGTTAAACCAACTAGAAA	ACCACTAATCCCCAGATTTT	+3 C to T
UBE3A-mpknot	CAGGGAAATACTGGAAACTC**AGG**	ATGAGTACCTAAGTTTCCAGTATTTCTCTCTCTCGGGTCAGGAGCCCCCCCCCTGAACCCAGGATAACCCTCAAAGTCGGGGGGCAACCC	ACCACTAATCCCCAGATTTT	+3 C to T
LSP1- xrRNA	CTGTGAGAAGGCAACACTGC**CGG**	CAGCCCCAGCCGACAGTGTTGCCTTCTCATCTCTCTCGCGTAACCTCCATCCGAGTTGCAAGAGAGGGAAACGCAGTCTC	CCTCTCTGGGCCCTTTGGGT	+3 C to T
LSP1-evopreQ1	CTGTGAGAAGGCAACACTGC**CGG**	CAGCCCCAGCCGACAGTGTTGCCTTCTCATCTCTCTCTTGACGCGGTTCTATCTAGTTACGCGTTAAACCAACTAGAAA	CCTCTCTGGGCCCTTTGGGT	+3 C to T
LSP1-mpknot	CTGTGAGAAGGCAACACTGC**CGG**	CAGCCCCAGCCGACAGTGTTGCCTTCTCATCTCTCTCGGGTCAGGAGCCCCCCCCCTGAACCCAGGATAACCCTCAAAGTCGGGGGGCAACCC	CCTCTCTGGGCCCTTTGGGT	+3 C to T
HEK3-D1-evopreQ1	GGCCCAGACTGAGCACGTGA**TGG**	TGGAGGAAGCAGGGCTTCCTTTCCTCTGCCATCTCGTGCTCAGTCTGTCTCTCTCCGCGGTTCTATCTAGTTACGCGTTAAACCAACTAGAA	GTCAACCAGTATCCCGGTGC	+1 T to A
RNF2-D1-evopreQ1	GTCATCTTAGTCATTACCTGA**GG**	AACGAACACCTCATGTAATGACTAAGATGTCATCTCTCGCGGTTCTATCTAGTTACGCGTTAAACCAACTAGAA	GTCAACCATTAAGCAAAACAT	+1 C to A

RUNX1-D1-evopreQ1	GCATTTTCAGGAGGAAGCGA**CGG**	TGTCTGAAGCAATCGCTTCCTCCTGAAAATAACTCTCTCGCGGTTCTATCTAGTTACGCGTTAAACCAACTAGAA	GATGAAGCACTGTGGGTACGA	+5 G to T
DNMT1-D1-evopreQ1	GATTCCTGGTGCCAGAAACA**TGG**	GTCACCACTGTTTCTGGCACCAGGACCTCTTCTCGCGGTTCTATCTAGTTACGCGTTAAACCAACTAGAA	GCCCTTCAGCTAAAATAAAGG	+5 G to T
HEK3-D1-hTR	GGCCCAGACTGAGCACGTGA**TGG**	TGGAGGAAGCAGGGCTTCCTTTCCTCTGCCATCTCGTGCTCAGTCTGTGGGTGGGCCTGGGAGGG	GTCAACCAGTATCCCGGTGC	+1 T to A
RNF2-D1-hTR	GTCATCTTAGTCATTACCTG**AGG**	AACGAACACCTCATGTAATGACTAAGATGGGGTGGGCCTGGGAGGG	GTCAACCATTAAGCAAAACAT	+1 C to A
RUNX1-D1-hTR	GCATTTTCAGGAGGAAGCGA**CGG**	TGTCTGAAGCAATCGCTTCCTCCTGAAAATGGGTGGGCCTGGGAGGG	GATGAAGCACTGTGGGTACGA	+5 G to T
DNMT1-D1-hTR	GATTCCTGGTGCCAGAAACA**TGG**	GTCACCACTGTTTCTGGCACCAGGAGGGTGGGCCTGGGAGGG	GCCCTTCAGCTAAAATAAAGG	+5 G to T
LSP1-RT17	CTGTGAGAAGGCAACACTGC**CGG**	CCCAGCCCCAGCCGACAGTGTTGCCTTCTCA	CCTCTCTGGGCCCTTTGGGT	+3 C to T
LSP1-RT16	CTGTGAGAAGGCAACACTGC**CGG**	CCAGCCCCAGCCGACAGTGTTGCCTTCTCA	CCTCTCTGGGCCCTTTGGGT	+3 C to T
LSP1-RT15	CTGTGAGAAGGCAACACTGC**CGG**	CAGCCCCAGCCGACAGTGTTGCCTTCTCA	CCTCTCTGGGCCCTTTGGGT	+3 C to T
LSP1-RT14	CTGTGAGAAGGCAACACTGC**CGG**	AGCCCCAGCCGACAGTGTTGCCTTCTCA	CCTCTCTGGGCCCTTTGGGT	+3 C to T
LSP1-RT13	CTGTGAGAAGGCAACACTGC**CGG**	GCCCCAGCCGACAGTGTTGCCTTCTCA	CCTCTCTGGGCCCTTTGGGT	+3 C to T
LSP1-RT12	CTGTGAGAAGGCAACACTGC**CGG**	CCCCAGCCGACAGTGTTGCCTTCTCA	CCTCTCTGGGCCCTTTGGGT	+3 C to T
LSP1-PBS16	CTGTGAGAAGGCAACACTGC**CGG**	CAGCCCCAGCCGACAGTGTTGCCTTCTCACA	CCTCTCTGGGCCCTTTGGGT	+3 C to T
LSP1-PBS15	CTGTGAGAAGGCAACACTGC**CGG**	CAGCCCCAGCCGACAGTGTTGCCTTCTCAC	CCTCTCTGGGCCCTTTGGGT	+3 C to T
LSP1-PBS14	CTGTGAGAAGGCAACACTGC**CGG**	CAGCCCCAGCCGACAGTGTTGCCTTCTCA	CCTCTCTGGGCCCTTTGGGT	+3 C to T
LSP1-PBS13	CTGTGAGAAGGCAACACTGC**CGG**	CAGCCCCAGCCGACAGTGTTGCCTTCTC	CCTCTCTGGGCCCTTTGGGT	+3 C to T
LSP1-PBS12	CTGTGAGAAGGCAACACTGC**CGG**	CAGCCCCAGCCGACAGTGTTGCCTTCT	CCTCTCTGGGCCCTTTGGGT	+3 C to T
LSP1-G4-RT17	CTGTGAGAAGGCAACACTGC**CGG**	CCCAGCCCCAGCCGACAGTGTTGCCTTCTCAGGGTGGGCCTGGGAGGG	CCTCTCTGGGCCCTTTGGGT	+3 C to T
LSP1-G4-RT16	CTGTGAGAAGGCAACACTGC**CGG**	CCAGCCCCAGCCGACAGTGTTGCCTTCTCAGGGTGGGCCTGGGAGGG	CCTCTCTGGGCCCTTTGGGT	+3 C to T
LSP1-G4-RT15	CTGTGAGAAGGCAACACTGC**CGG**	CAGCCCCAGCCGACAGTGTTGCCTTCTCAGGGTGGGCCTGGGAGGG	CCTCTCTGGGCCCTTTGGGT	+3 C to T
LSP1-G4-RT14	CTGTGAGAAGGCAACACTGC**CGG**	AGCCCCAGCCGACAGTGTTGCCTTCTCAGGGTGGGCCTGGGAGGG	CCTCTCTGGGCCCTTTGGGT	+3 C to T
LSP1-G4-RT13	CTGTGAGAAGGCAACACTGC**CGG**	GCCCCAGCCGACAGTGTTGCCTTCTCAGGGTGGGCCTGGGAGGG	CCTCTCTGGGCCCTTTGGGT	+3 C to T
LSP1-G4-RT12	CTGTGAGAAGGCAACACTGC**CGG**	CCCCAGCCGACAGTGTTGCCTTCTCAGGGTGGGCCTGGGAGGG	CCTCTCTGGGCCCTTTGGGT	+3 C to T
LSP1-G4-PBS16	CTGTGAGAAGGCAACACTGC**CGG**	CAGCCCCAGCCGACAGTGTTGCCTTCTCACAGGGTGGGCCTGGGAGGG	CCTCTCTGGGCCCTTTGGGT	+3 C to T
LSP1-G4-PBS15	CTGTGAGAAGGCAACACTGC**CGG**	CAGCCCCAGCCGACAGTGTTGCCTTCTCACGGGTGGGCCTGGGAGGG	CCTCTCTGGGCCCTTTGGGT	+3 C to T
LSP1-G4-PBS14	CTGTGAGAAGGCAACACTGC**CGG**	CAGCCCCAGCCGACAGTGTTGCCTTCTCAGGGTGGGCCTGGGAGGG	CCTCTCTGGGCCCTTTGGGT	+3 C to T
LSP1-G4-PBS13	CTGTGAGAAGGCAACACTGC**CGG**	CAGCCCCAGCCGACAGTGTTGCCTTCTCGGGTGGGCCTGGGAGGG	CCTCTCTGGGCCCTTTGGGT	+3 C to T
LSP1-G4-PBS12	CTGTGAGAAGGCAACACTGC**CGG**	CAGCCCCAGCCGACAGTGTTGCCTTCTGGGTGGGCCTGGGAGGG	CCTCTCTGGGCCCTTTGGGT	+3 C to T
SEC61B-RT17	ACTGATTGCTTTCAAAGTTC**TGG**	CTGTGTTCATACCAGAACTTTGAAAGCAAT	GCCCTGCTACATGACGGAGT	+7 C to T
SEC61B-RT16	ACTGATTGCTTTCAAAGTTC**TGG**	TGTGTTCATACCAGAACTTTGAAAGCAAT	GCCCTGCTACATGACGGAGT	+7 C to T
SEC61B-RT15	ACTGATTGCTTTCAAAGTTC**TGG**	GTGTTCATACCAGAACTTTGAAAGCAAT	GCCCTGCTACATGACGGAGT	+7 C to T

SEC61B-RT14	ACTGATTGCTTTCAAAGTT **CTGG**	TGTTCATACCAGAACTTTGAAAGCAAT	GCCCTGCTACATG ACGGAGT	+7 C to T
SEC61B-RT13	ACTGATTGCTTTCAAAGTT **CTGG**	GTTCATACCAGAACTTTGAAAGCAAT	GCCCTGCTACATG ACGGAGT	+7 C to T
SEC61B-RT12	ACTGATTGCTTTCAAAGTT **CTGG**	TTCATACCAGAACTTTGAAAGCAAT	GCCCTGCTACATG ACGGAGT	+7 C to T
SEC61B-PBS16	ACTGATTGCTTTCAAAGTT **CTGG**	TGTTCATACCAGAACTTTGAAAGCAATCAG	GCCCTGCTACATG ACGGAGT	+7 C to T
SEC61B-PBS15	ACTGATTGCTTTCAAAGTT **CTGG**	TGTTCATACCAGAACTTTGAAAGCAATCA	GCCCTGCTACATG ACGGAGT	+7 C to T
SEC61B-PBS14	ACTGATTGCTTTCAAAGTT **CTGG**	TGTTCATACCAGAACTTTGAAAGCAATC	GCCCTGCTACATG ACGGAGT	+7 C to T
SEC61B-PBS13	ACTGATTGCTTTCAAAGTT **CTGG**	TGTTCATACCAGAACTTTGAAAGCAAT	GCCCTGCTACATG ACGGAGT	+7 C to T
SEC61B-PBS12	ACTGATTGCTTTCAAAGTT **CTGG**	TGTTCATACCAGAACTTTGAAAGCAA	GCCCTGCTACATG ACGGAGT	+7 C to T
SEC61B-G4-RT17	ACTGATTGCTTTCAAAGTT **CTGG**	CTGTGTTCATACCAGAACTTTGAAAGCAATG GGTGGGCCTGGGAGGG	GCCCTGCTACATG ACGGAGT	+7 C to T
SEC61B-G4-RT16	ACTGATTGCTTTCAAAGTT **CTGG**	TGTGTTCATACCAGAACTTTGAAAGCAATGG GTGGGCCTGGGAGGG	GCCCTGCTACATG ACGGAGT	+7 C to T
SEC61B-G4-RT15	ACTGATTGCTTTCAAAGTT **CTGG**	GTGTTCATACCAGAACTTTGAAAGCAATGGG TGGGCCTGGGAGGG	GCCCTGCTACATG ACGGAGT	+7 C to T
SEC61B-G4-RT14	ACTGATTGCTTTCAAAGTT **CTGG**	TGTTCATACCAGAACTTTGAAAGCAATGGGT GGGCCTGGGAGGG	GCCCTGCTACATG ACGGAGT	+7 C to T
SEC61B-G4-RT13	ACTGATTGCTTTCAAAGTT **CTGG**	GTTCATACCAGAACTTTGAAAGCAATGGGTG GGCCTGGGAGGG	GCCCTGCTACATG ACGGAGT	+7 C to T
SEC61B-G4-RT12	ACTGATTGCTTTCAAAGTT **CTGG**	TTCATACCAGAACTTTGAAAGCAATGGGTGG GCCTGGGAGGG	GCCCTGCTACATG ACGGAGT	+7 C to T
SEC61B-G4-PBS16	ACTGATTGCTTTCAAAGTT **CTGG**	TGTTCATACCAGAACTTTGAAAGCAATCAGG GGTGGGCCTGGGAGGG	GCCCTGCTACATG ACGGAGT	+7 C to T
SEC61B-G4-PBS15	ACTGATTGCTTTCAAAGTT **CTGG**	TGTTCATACCAGAACTTTGAAAGCAATCAGG GTGGGCCTGGGAGGG	GCCCTGCTACATG ACGGAGT	+7 C to T
SEC61B-G4-PBS14	ACTGATTGCTTTCAAAGTT **CTGG**	TGTTCATACCAGAACTTTGAAAGCAATCGGG TGGGCCTGGGAGGG	GCCCTGCTACATG ACGGAGT	+7 C to T
SEC61B-G4-PBS13	ACTGATTGCTTTCAAAGTT **CTGG**	TGTTCATACCAGAACTTTGAAAGCAATGGGT GGGCCTGGGAGGG	GCCCTGCTACATG ACGGAGT	+7 C to T
SEC61B-G4-PBS12	ACTGATTGCTTTCAAAGTT **CTGG**	TGTTCATACCAGAACTTTGAAAGCAAGGGTG GGCCTGGGAGGG	GCCCTGCTACATG ACGGAGT	+7 C to T
RUNX1-RT17	GCATTTTCAGGAGGAAGC GA**TGG**	GCTGTCTGAAACCATCGCTTCCTCCTGAAAA T	ATGAAGCACTGTG GGTACGA	+7 C to T
RUNX1-RT16	GCATTTTCAGGAGGAAGC GA**TGG**	CTGTCTGAAACCATCGCTTCCTCCTGAAAAT	ATGAAGCACTGTG GGTACGA	+7 C to T
RUNX1-RT15	GCATTTTCAGGAGGAAGC GA**TGG**	TGTCTGAAACCATCGCTTCCTCCTGAAAAT	ATGAAGCACTGTG GGTACGA	+7 C to T
RUNX1-RT14	GCATTTTCAGGAGGAAGC GA**TGG**	GTCTGAAACCATCGCTTCCTCCTGAAAAT	ATGAAGCACTGTG GGTACGA	+7 C to T
RUNX1-RT13	GCATTTTCAGGAGGAAGC GA**TGG**	TCTGAAACCATCGCTTCCTCCTGAAAAT	ATGAAGCACTGTG GGTACGA	+7 C to T
RUNX1-RT12	GCATTTTCAGGAGGAAGC GA**TGG**	CTGAAACCATCGCTTCCTCCTGAAAAT	ATGAAGCACTGTG GGTACGA	+7 C to T
RUNX1-PBS16	GCATTTTCAGGAGGAAGC GA**TGG**	TGTCTGAAACCATCGCTTCCTCCTGAAAATG	ATGAAGCACTGTG GGTACGA	+7 C to T
RUNX1-PBS15	GCATTTTCAGGAGGAAGC GA**TGG**	TGTCTGAAACCATCGCTTCCTCCTGAAAAT	ATGAAGCACTGTG GGTACGA	+7 C to T
RUNX1-PBS14	GCATTTTCAGGAGGAAGC GA**TGG**	TGTCTGAAACCATCGCTTCCTCCTGAAAA	ATGAAGCACTGTG GGTACGA	+7 C to T
RUNX1-PBS13	GCATTTTCAGGAGGAAGC GA**TGG**	TGTCTGAAACCATCGCTTCCTCCTGAAA	ATGAAGCACTGTG GGTACGA	+7 C to T
RUNX1-PBS12	GCATTTTCAGGAGGAAGC GA**TGG**	TGTCTGAAACCATCGCTTCCTCCTGAA	ATGAAGCACTGTG GGTACGA	+7 C to T
RUNX1-G4-RT17	GCATTTTCAGGAGGAAGC GA**TGG**	GCTGTCTGAAACCATCGCTTCCTCCTGAAAA TGGGTGGGCCTGGGAGGG	ATGAAGCACTGTG GGTACGA	+7 C to T
RUNX1-G4-RT16	GCATTTTCAGGAGGAAGC GA**TGG**	CTGTCTGAAACCATCGCTTCCTCCTGAAAAT GGGTGGGCCTGGGAGGG	ATGAAGCACTGTG GGTACGA	+7 C to T
RUNX1-G4-RT15	GCATTTTCAGGAGGAAGC GA**TGG**	TGTCTGAAACCATCGCTTCCTCCTGAAAATG GGTGGGCCTGGGAGGG	ATGAAGCACTGTG GGTACGA	+7 C to T

RUNX1-G4-RT14	GCATTTTCAGGAGGAAGCGA**TGG**	GTCTGAAACCATCGCTTCCTCCTGAAAATGGGTGGGCCTGGGAGGG	ATGAAGCACTGTGGGTACGA	+7 C to T
RUNX1-G4-RT13	GCATTTTCAGGAGGAAGCGA**TGG**	TCTGAAACCATCGCTTCCTCCTGAAAATGGGTGGGCCTGGGAGGG	ATGAAGCACTGTGGGTACGA	+7 C to T
RUNX1-G4-RT12	GCATTTTCAGGAGGAAGCGA**TGG**	CTGAAACCATCGCTTCCTCCTGAAAATGGGTGGGCCTGGGAGGG	ATGAAGCACTGTGGGTACGA	+7 C to T
RUNX1-PBS16	GCATTTTCAGGAGGAAGCGA**TGG**	TGTCTGAAACCATCGCTTCCTCCTGAAAATGGGGTGGGCCTGGGAGGG	ATGAAGCACTGTGGGTACGA	+7 C to T
RUNX1-G4-PBS15	GCATTTTCAGGAGGAAGCGA**TGG**	TGTCTGAAACCATCGCTTCCTCCTGAAAATGGGTGGGCCTGGGAGGG	ATGAAGCACTGTGGGTACGA	+7 C to T
RUNX1-G4-PBS14	GCATTTTCAGGAGGAAGCGA**TGG**	TGTCTGAAACCATCGCTTCCTCCTGAAAAGGGTGGGCCTGGGAGGG	ATGAAGCACTGTGGGTACGA	+7 C to T
RUNX1-G4-PBS13	GCATTTTCAGGAGGAAGCGA**TGG**	TGTCTGAAACCATCGCTTCCTCCTGAAAGGGTGGGCCTGGGAGGG	ATGAAGCACTGTGGGTACGA	+7 C to T
RUNX1-G4-PBS12	GCATTTTCAGGAGGAAGCGA**TGG**	TGTCTGAAACCATCGCTTCCTCCTGAAGGGTGGGCCTGGGAGGG	ATGAAGCACTGTGGGTACGA	+7 C to T
SHANK3-RT17	GTATCTCGAGTCGGTGGACG**TGG**	GGCCACACCCTCCACGACCACCGACTCGAG	AGAACGACCTCACCTCGATG	+1 A to T
SHANK3-RT16	GTATCTCGAGTCGGTGGACG**TGG**	GCCACACCCTCCACGACCACCGACTCGAG	AGAACGACCTCACCTCGATG	+1 A to T
SHANK3-RT15	GTATCTCGAGTCGGTGGACG**TGG**	CCACACCCTCCACGACCACCGACTCGAG	AGAACGACCTCACCTCGATG	+1 A to T
SHANK3-RT14	GTATCTCGAGTCGGTGGACG**TGG**	CACACCCTCCACGACCACCGACTCGAG	AGAACGACCTCACCTCGATG	+1 A to T
SHANK3-RT13	GTATCTCGAGTCGGTGGACG**TGG**	ACACCCTCCACGACCACCGACTCGAG	AGAACGACCTCACCTCGATG	+1 A to T
SHANK3-RT12	GTATCTCGAGTCGGTGGACG**TGG**	CACCCTCCACGACCACCGACTCGAG	AGAACGACCTCACCTCGATG	+1 A to T
SHANK3-PBS16	GTATCTCGAGTCGGTGGACG**TGG**	ACACCCTCCACGACCACCGACTCGAGATA	AGAACGACCTCACCTCGATG	+1 A to T
SHANK3-PBS15	GTATCTCGAGTCGGTGGACG**TGG**	ACACCCTCCACGACCACCGACTCGAGAT	AGAACGACCTCACCTCGATG	+1 A to T
SHANK3-PBS14	GTATCTCGAGTCGGTGGACG**TGG**	ACACCCTCCACGACCACCGACTCGAGA	AGAACGACCTCACCTCGATG	+1 A to T
SHANK3-PBS13	GTATCTCGAGTCGGTGGACG**TGG**	ACACCCTCCACGACCACCGACTCGAG	AGAACGACCTCACCTCGATG	+1 A to T
SHANK3-PBS12	GTATCTCGAGTCGGTGGACG**TGG**	ACACCCTCCACGACCACCGACTCGA	AGAACGACCTCACCTCGATG	+1 A to T
SHANK3-G4-RT17	GTATCTCGAGTCGGTGGACG**TGG**	GGCCACACCCTCCACGACCACCGACTCGAGGGGTGGGCCTGGGAGGG	AGAACGACCTCACCTCGATG	+1 A to T
SHANK3-G4-RT16	GTATCTCGAGTCGGTGGACG**TGG**	GCCACACCCTCCACGACCACCGACTCGAGGGGTGGGCCTGGGAGGG	AGAACGACCTCACCTCGATG	+1 A to T
SHANK3-G4-RT15	GTATCTCGAGTCGGTGGACG**TGG**	CCACACCCTCCACGACCACCGACTCGAGGGGTGGGCCTGGGAGGG	AGAACGACCTCACCTCGATG	+1 A to T
SHANK3-G4-RT14	GTATCTCGAGTCGGTGGACG**TGG**	CACACCCTCCACGACCACCGACTCGAGGGTGGGCCTGGGAGGG	AGAACGACCTCACCTCGATG	+1 A to T
SHANK3-G4-RT13	GTATCTCGAGTCGGTGGACG**TGG**	ACACCCTCCACGACCACCGACTCGAGGGGTGGGCCTGGGAGGG	AGAACGACCTCACCTCGATG	+1 A to T
SHANK3-G4-RT12	GTATCTCGAGTCGGTGGACG**TGG**	CACCCTCCACGACCACCGACTCGAGGGGTGGGCCTGGGAGGG	AGAACGACCTCACCTCGATG	+1 A to T
SHANK3-PBS16	GTATCTCGAGTCGGTGGACG**TGG**	ACACCCTCCACGACCACCGACTCGAGATAGGGTGGGCCTGGGAGGG	AGAACGACCTCACCTCGATG	+1 A to T
SHANK3-G4-PBS15	GTATCTCGAGTCGGTGGACG**TGG**	ACACCCTCCACGACCACCGACTCGAGATGGGTGGGCCTGGGAGGG	AGAACGACCTCACCTCGATG	+1 A to T
SHANK3-G4-PBS14	GTATCTCGAGTCGGTGGACG**TGG**	ACACCCTCCACGACCACCGACTCGAGAGGGTGGGCCTGGGAGGG	AGAACGACCTCACCTCGATG	+1 A to T
SHANK3-G4-PBS13	GTATCTCGAGTCGGTGGACG**TGG**	ACACCCTCCACGACCACCGACTCGAGGGGTGGGCCTGGGAGGG	AGAACGACCTCACCTCGATG	+1 A to T
SHANK3-G4-PBS12	GTATCTCGAGTCGGTGGACG**TGG**	ACACCCTCCACGACCACCGACTCGAGGGTGGGCCTGGGAGGG	AGAACGACCTCACCTCGATG	+1 A to T

The PAM in each target sequence is shown in bold. The PBS sequence are shown in blue.

The RT template are shown in red. The modifications are shown in green.

Supplementary Table S3. Primers used for cell genomic DNA amplification and targeted deep sequencing.

NO.	Forward Primer	Reverse Primer
JD-FANCF-F	TGCCCAGAGTCAAGGAACAC	CGACCAAAGCGCCGATGGAT
JD-RUNX1-T1-F	AATAGGGCTTGGGGAGTCCC	CAAAGTTCTCACGCACCGAC
JD-RNF2-F	CCTGATCACCTCCCAAAGTC	CTGTAAAGTCCATGGTTGGT
JD-SEC61B-T1-F	CATACCCCACAGATGGAATG	CCTATCTCATTGCTGCCTGC
JD-LSP1-F	CACTGAACCCTGGGTAGTGA	GACACAGACAGCCAGAAGCT
JD-SHANK3-T2-F	ATGTCTAGGATCCTTGGGCC	AACCTTCATGACGAGGCGGT
JD-SHANK3-T1-F	AAGGAGGTGCGCTTCGTGGT	ACTAAGCAATGTACAGGGCG
JD-UBE3A-T1-F	GATGAAGTGACCATGGCAGA	GCAATGACAGGGATAGCTAA
seq-LSP1-hTR-F	CTCAAGAGGTCCCCAAATGC	CCATCACACAGGGAGGAGGT
seq-LSP1-TERRA-F	AGTCAACTCAAGAGGTCCCCAAATGC	CCATCACACAGGGAGGAGGT
seq-LSP1-MT3-F	AGTTCCACTCAAGAGGTCCCCAAATGC	CCATCACACAGGGAGGAGGT
seq-LSP1-NRAS-F	ATGTCACGCTCAAGAGGTCCCCAAATGC	CCATCACACAGGGAGGAGGT
seq-LSP1-HS-F	CACCGGTCTCAAGAGGTCCCCAAATGC	CCATCACACAGGGAGGAGGT
seq-LSP1-NS-F	CACGATGCTCAAGAGGTCCCCAAATGC	CCATCACACAGGGAGGAGGT
seq-LSP1-F	GAGTGGCTCAAGAGGTCCCCAAATGC	CCATCACACAGGGAGGAGGT
seq-SEC61B-hTR-F	AGTTCCCAGGCTACCACGTA	AACCACGGTCTTTACTCCAC
seq-SEC61B-TERRA-F	AGTCAAAGTTCCCAGGCTACCACGTA	AACCACGGTCTTTACTCCAC
seq-SEC61B-MT3-F	AGTTCCAAGTTCCCAGGCTACCACGTA	AACCACGGTCTTTACTCCAC
seq-SEC61B-NRAS-F	ATGTCACGAGTTCCCAGGCTACCACGTA	AACCACGGTCTTTACTCCAC
seq-SEC61B-HS-F	CACCGGTAGTTCCCAGGCTACCACGTA	AACCACGGTCTTTACTCCAC
seq-SEC61B-NS-F	CACGATGAGTTCCCAGGCTACCACGTA	AACCACGGTCTTTACTCCAC
seq-SEC61B-F	GAGTGGAGTTCCCAGGCTACCACGTA	AACCACGGTCTTTACTCCAC
seq-RNF2-hTR-F	ATTTCCAGCAATGTCTCAGG	ACAGAAGTCAGGAATGCTTG
seq-RNF2-TERRA-F	AGTCAAATTTCCAGCAATGTCTCAGG	ACAGAAGTCAGGAATGCTTG
seq-RNF2-MT3-F	AGTTCCAATTTCCAGCAATGTCTCAGG	ACAGAAGTCAGGAATGCTTG
seq-RNF2-NRAS-F	ATGTCACGATTTCCAGCAATGTCTCAGG	ACAGAAGTCAGGAATGCTTG
seq-RNF2-HS-F	CACCGGTATTTCCAGCAATGTCTCAGG	ACAGAAGTCAGGAATGCTTG
seq-RNF2-NS-F	CACGATGATTTCCAGCAATGTCTCAGG	ACAGAAGTCAGGAATGCTTG
seq-RNF2-F	GAGTGGATTTCCAGCAATGTCTCAGG	ACAGAAGTCAGGAATGCTTG
seq-RUNX1-hTR-F	GTGAGGCTGAAACAGTGACC	TGCACCGAGGTGAAACAAGC

seq-RUNX1-TERRA-F	AGTCAAGTGAGGCTGAAACAGTGACC	TGCACCGAGGTGAAACAAGC
seq-RUNX1-MT3-F	AGTTCCAGTGAGGCTGAAACAGTGACC	TGCACCGAGGTGAAACAAGC
seq-RUNX1-NRAS-F	ATGTCACGGTGAGGCTGAAACAGTGACC	TGCACCGAGGTGAAACAAGC
seq-RUNX1-HS-F	CACCGGTGTGAGGCTGAAACAGTGACC	TGCACCGAGGTGAAACAAGC
seq-RUNX1-NS-F	CACGATGGTGAGGCTGAAACAGTGACC	TGCACCGAGGTGAAACAAGC
seq-RUNX1-F	GAGTGGGTGAGGCTGAAACAGTGACC	TGCACCGAGGTGAAACAAGC
seq-FANCF-hTR-F	GGATGTTCCAATCAGTACGC	ATGCCGACCAAAGCGCCGAT
seq-FANCF-TERRA-F	AGTCAAGGATGTTCCAATCAGTACGC	ATGCCGACCAAAGCGCCGAT
seq-FANCF-MT3-F	AGTTCCAGGATGTTCCAATCAGTACGC	ATGCCGACCAAAGCGCCGAT
seq-FANCF-NRAS-F	ATGTCACGGGATGTTCCAATCAGTACGC	ATGCCGACCAAAGCGCCGAT
seq-FANCF-HS-F	CACCGGTGGATGTTCCAATCAGTACGC	ATGCCGACCAAAGCGCCGAT
seq-FANCF-NS-F	CACGATGGGATGTTCCAATCAGTACGC	ATGCCGACCAAAGCGCCGAT
seq-FANCF-F	GAGTGGGGATGTTCCAATCAGTACGC	ATGCCGACCAAAGCGCCGAT
seq-UBE3A-hTR-F	TCATAGAGACAACTCTGGGT	TTCTGGCCACTCTTATATTC
seq-UBE3A-TERRA-F	AGTCAATCATAGAGACAACTCTGGGT	TTCTGGCCACTCTTATATTC
seq-UBE3A-MT3-F	AGTTCCATCATAGAGACAACTCTGGGT	TTCTGGCCACTCTTATATTC
seq-UBE3A-NRAS-F	ATGTCACGTCATAGAGACAACTCTGGGT	TTCTGGCCACTCTTATATTC
seq-UBE3A-HS-F	CACCGGTTCATAGAGACAACTCTGGGT	TTCTGGCCACTCTTATATTC
seq-UBE3A-NS-F	CACGATGTCATAGAGACAACTCTGGGT	TTCTGGCCACTCTTATATTC
seq-UBE3A-F	GAGTGGTCATAGAGACAACTCTGGGT	TTCTGGCCACTCTTATATTC
seq-SHANKS-T1-hTR-F	TTCCAGCAGAAGCCGCTGCA	CAGCTGCCGTCCAGCTGCCT
seq-SHANKS-T1-TERRA-F	AGTCAATTCCAGCAGAAGCCGCTGCA	CAGCTGCCGTCCAGCTGCCT
seq-SHANKS-T1-MT3-F	AGTTCCATTCCAGCAGAAGCCGCTGCA	CAGCTGCCGTCCAGCTGCCT
seq-SHANKS-T1-NRAS-F	ATGTCACGTTCCAGCAGAAGCCGCTGCA	CAGCTGCCGTCCAGCTGCCT
seq-SHANKS-T1-HS-F	CACCGGTTTCCAGCAGAAGCCGCTGCA	CAGCTGCCGTCCAGCTGCCT
seq-SHANKS-T1-NS-F	CACGATGTTCCAGCAGAAGCCGCTGCA	CAGCTGCCGTCCAGCTGCCT
seq-SHANKS-T1-F	GAGTGGTTCCAGCAGAAGCCGCTGCA	CAGCTGCCGTCCAGCTGCCT
seq-SHANK3-T2-hTR-F	CTAGCTGGTGAAGCGCCTTC	ACGTCCACCACAGGGCTCAG
seq-SHANK3-T2-TERRA-F	AGTCAACTAGCTGGTGAAGCGCCTTC	ACGTCCACCACAGGGCTCAG
seq-SHANK3-T2-MT3-F	AGTTCCACTAGCTGGTGAAGCGCCTTC	ACGTCCACCACAGGGCTCAG
seq-SHANK3-T2-NRAS-F	ATGTCACGCTAGCTGGTGAAGCGCCTTC	ACGTCCACCACAGGGCTCAG
seq-SHANK3-T2-HS-F	CACCGGTCTAGCTGGTGAAGCGCCTTC	ACGTCCACCACAGGGCTCAG
seq-SHANK3-T2-NS-F	CACGATGCTAGCTGGTGAAGCGCCTTC	ACGTCCACCACAGGGCTCAG

seq-SHANK3-T2-F	GAGTGGCTAGCTGGTGAAGCGCCTTC	ACGTCCACCACAGGGCTCAG
seq-LSP1-F	CTCAAGAGGTCCCCAAATGC	CCATCACACAGGGAGGAGGT
seq-LSP1-G4-F	AGTCAACTCAAGAGGTCCCCAAATGC	CCATCACACAGGGAGGAGGT
seq-LSP1-del-F	AGTTCCACTCAAGAGGTCCCCAAATGC	CCATCACACAGGGAGGAGGT
seq-LSP1-G4-del--F	ATGTCACGCTCAAGAGGTCCCCAAATGC	CCATCACACAGGGAGGAGGT
seq-LSP1-ins-F	CACCGGTCTCAAGAGGTCCCCAAATGC	CCATCACACAGGGAGGAGGT
seq-LSP1-G4-ins-F	CACGATGCTCAAGAGGTCCCCAAATGC	CCATCACACAGGGAGGAGGT
seq-LSP1-untreated-F	GAGTGGCTCAAGAGGTCCCCAAATGC	CCATCACACAGGGAGGAGGT
seq-SEC61B-F	AGTTCCCAGGCTACCACGTA	AACCACGGTCTTTACTCCAC
seq-SEC61B-G4-F	AGTCAAAGTTCCCAGGCTACCACGTA	AACCACGGTCTTTACTCCAC
seq-SEC61B-del- F	AGTTCCAAGTTCCCAGGCTACCACGTA	AACCACGGTCTTTACTCCAC
seq-SEC61B-G4-del-F	ATGTCACGAGTTCCCAGGCTACCACGTA	AACCACGGTCTTTACTCCAC
seq-SEC61B-ins-F	CACCGGTAGTTCCCAGGCTACCACGTA	AACCACGGTCTTTACTCCAC
seq-SEC61B-G4-ins-F	CACGATGAGTTCCCAGGCTACCACGTA	AACCACGGTCTTTACTCCAC
seq-SEC61B-untreated-F	GAGTGGAGTTCCCAGGCTACCACGTA	AACCACGGTCTTTACTCCAC
seq-RNF2-F	ATTTCCAGCAATGTCTCAGG	ACAGAAGTCAGGAATGCTTG
seq-RNF2-G4-F	AGTCAAATTTCCAGCAATGTCTCAGG	ACAGAAGTCAGGAATGCTTG
seq-RNF2-del-F	AGTTCCAATTTCCAGCAATGTCTCAGG	ACAGAAGTCAGGAATGCTTG
seq-RNF2-G4-del-F	ATGTCACGATTTCCAGCAATGTCTCAGG	ACAGAAGTCAGGAATGCTTG
seq-RNF2-ins-F	CACCGGTATTTCCAGCAATGTCTCAGG	ACAGAAGTCAGGAATGCTTG
seq-RNF2-G4-ins-F	CACGATGATTTCCAGCAATGTCTCAGG	ACAGAAGTCAGGAATGCTTG
seq-RNF2-untreated-F	GAGTGGATTTCCAGCAATGTCTCAGG	ACAGAAGTCAGGAATGCTTG
seq-RUNX1-F	GTGAGGCTGAAACAGTGACC	TGCACCGAGGTGAAACAAGC
seq-RUNX1-G4-F	AGTCAAGTGAGGCTGAAACAGTGACC	TGCACCGAGGTGAAACAAGC
seq-RUNX1-del-F	AGTTCCAGTGAGGCTGAAACAGTGACC	TGCACCGAGGTGAAACAAGC
seq-RUNX1-G4-del-F	ATGTCACGGTGAGGCTGAAACAGTGACC	TGCACCGAGGTGAAACAAGC
seq-RUNX1-ins-F	CACCGGTGTGAGGCTGAAACAGTGACC	TGCACCGAGGTGAAACAAGC
seq-RUNX1-G4-ins-F	CACGATGGTGAGGCTGAAACAGTGACC	TGCACCGAGGTGAAACAAGC
seq-RUNX1-untreated-F	GAGTGGGTGAGGCTGAAACAGTGACC	TGCACCGAGGTGAAACAAGC
seq-FANCF-F	GGATGTTCCAATCAGTACGC	ATGCCGACCAAAGCGCCGAT
seq-FANCF-G4-F	AGTCAAGGATGTTCCAATCAGTACGC	ATGCCGACCAAAGCGCCGAT
seq-FANCF-del-F	AGTTCCAGGATGTTCCAATCAGTACGC	ATGCCGACCAAAGCGCCGAT
seq-FANCF-G4-del-F	ATGTCACGGGATGTTCCAATCAGTACGC	ATGCCGACCAAAGCGCCGAT

seq-FANCF-ins-F	CACCGGTGGATGTTCCAATCAGTACGC	ATGCCGACCAAAGCGCCGAT
seq-FANCF-G4-ins-F	CACGATGGGATGTTCCAATCAGTACGC	ATGCCGACCAAAGCGCCGAT
seq-FANCF-untreated-F	GAGTGGGGATGTTCCAATCAGTACGC	ATGCCGACCAAAGCGCCGAT
seq-UBE3A-F	TCATAGAGACAACTCTGGGT	TTCTGGCCACTCTTATATTC
seq-UBE3A-F	TCATAGAGACAACTCTGGGT	TTCTGGCCACTCTTATATTC
seq-UBE3A-G4-F	AGTCAATCATAGAGACAACTCTGGGT	TTCTGGCCACTCTTATATTC
seq-UBE3A-del-F	AGTTCCATCATAGAGACAACTCTGGGT	TTCTGGCCACTCTTATATTC
seq-UBE3A-G4-del-F	ATGTCACGTCATAGAGACAACTCTGGGT	TTCTGGCCACTCTTATATTC
seq-UBE3A-ins-F	CACCGGTTCATAGAGACAACTCTGGGT	TTCTGGCCACTCTTATATTC
seq-UBE3A-G4-ins-F	CACGATGTCATAGAGACAACTCTGGGT	TTCTGGCCACTCTTATATTC
seq-UBE3A-untreated-F	GAGTGGTCATAGAGACAACTCTGGGT	TTCTGGCCACTCTTATATTC
seq-SHANKS-T1-F	TTCCAGCAGAAGCCGCTGCA	CAGCTGCCGTCCAGCTGCCT
seq-SHANKS-T1-G4-F	AGTCAATTCCAGCAGAAGCCGCTGCA	CAGCTGCCGTCCAGCTGCCT
seq-SHANKS-T1-del-F	AGTTCCATTCCAGCAGAAGCCGCTGCA	CAGCTGCCGTCCAGCTGCCT
seq-SHANKS-T1-G4-del-F	ATGTCACGTTCCAGCAGAAGCCGCTGCA	CAGCTGCCGTCCAGCTGCCT
seq-SHANKS-T1-ins-F	CACCGGTTTCCAGCAGAAGCCGCTGCA	CAGCTGCCGTCCAGCTGCCT
seq-SHANKS-T1-G4-ins-F	CACGATGTTCCAGCAGAAGCCGCTGCA	CAGCTGCCGTCCAGCTGCCT
seq-SHANKS-T1-untreated-F	GAGTGGTTCCAGCAGAAGCCGCTGCA	CAGCTGCCGTCCAGCTGCCT
seq-SHANK3-T2-F	CTAGCTGGTGAAGCGCCTTC	ACGTCCACCACAGGGCTCAG
seq-SHANK3-T2-G4-F	AGTCAACTAGCTGGTGAAGCGCCTTC	ACGTCCACCACAGGGCTCAG
seq-SHANK3-T2-del-F	AGTTCCACTAGCTGGTGAAGCGCCTTC	ACGTCCACCACAGGGCTCAG
seq-SHANK3-T2-G4-del-F	ATGTCACGCTAGCTGGTGAAGCGCCTTC	ACGTCCACCACAGGGCTCAG
seq-SHANK3-T2-ins-F	CACCGGTCTAGCTGGTGAAGCGCCTTC	ACGTCCACCACAGGGCTCAG
seq-SHANK3-T2-G4-ins-F	CACGATGCTAGCTGGTGAAGCGCCTTC	ACGTCCACCACAGGGCTCAG
seq-SHANK3-T2-untreated-F	GAGTGGCTAGCTGGTGAAGCGCCTTC	ACGTCCACCACAGGGCTCAG
seq-LSP1-PBS16-F	ATGTCACGCTCAAGAGGTCCCCAAATGC	CCATCACACAGGGAGGAGGT
seq-LSP1-PBS15-F	CACCGGTCTCAAGAGGTCCCCAAATGC	CCATCACACAGGGAGGAGGT
seq-LSP1-PBS14-F	CACGATGCTCAAGAGGTCCCCAAATGC	CCATCACACAGGGAGGAGGT
seq-LSP1-PBS13-F	GAGTGGCTCAAGAGGTCCCCAAATGC	CCATCACACAGGGAGGAGGT
seq-LSP1-PBS12-F	GTAGAGCCTCAAGAGGTCCCCAAATGC	CCATCACACAGGGAGGAGGT
seq-LSP1-RT17-F	GTGGCCCTCAAGAGGTCCCCAAATGC	CCATCACACAGGGAGGAGGT
seq-LSP1-RT16-F	TCCCGACTCAAGAGGTCCCCAAATGC	CCATCACACAGGGAGGAGGT
seq-LSP1-RT15-F	GTCCGCACTCAAGAGGTCCCCAAATGC	CCATCACACAGGGAGGAGGT

seq-LSP1-RT14-F	ACTGATGCTCAAGAGGTCCCCAAATGC	CCATCACACAGGGAGGAGGT
seq-LSP1-RT13-F	ATTCCTCTCAAGAGGTCCCCAAATGC	CCATCACACAGGGAGGAGGT
seq-LSP1-RT12-F	GTGGCCCTCAAGAGGTCCCCAAATGC	CCATCACACAGGGAGGAGGT
seq-LSP1-PBS16-F	ATGTCACGCTCAAGAGGTCCCCAAATGC	CCATCACACAGGGAGGAGGT
seq-LSP1-PBS15-F	CACCGGTCTCAAGAGGTCCCCAAATGC	CCATCACACAGGGAGGAGGT
seq-LSP1-PBS14-F	CACGATGCTCAAGAGGTCCCCAAATGC	CCATCACACAGGGAGGAGGT
seq-LSP1-PBS13-F	CGGAATCCTCAAGAGGTCCCCAAATGC	CCATCACACAGGGAGGAGGT
seq-LSP1-PBS12-F	GAGTGGCTCAAGAGGTCCCCAAATGC	CCATCACACAGGGAGGAGGT
seq-LSP1-RT17-F	GTGGCCCTCAAGAGGTCCCCAAATGC	CCATCACACAGGGAGGAGGT
seq-LSP1-RT16-F	TCCCGACTCAAGAGGTCCCCAAATGC	CCATCACACAGGGAGGAGGT
seq-LSP1-RT15-F	CGGAATCCTCAAGAGGTCCCCAAATGC	CCATCACACAGGGAGGAGGT
seq-LSP1-RT14-F	GTCCGCACTCAAGAGGTCCCCAAATGC	CCATCACACAGGGAGGAGGT
seq-LSP1-RT13-F	ACTGATGCTCAAGAGGTCCCCAAATGC	CCATCACACAGGGAGGAGGT
seq-LSP1-RT12-F	ATTCCTCTCAAGAGGTCCCCAAATGC	CCATCACACAGGGAGGAGGT
seq-SEC61B-PBS16-F	ATGTCACGAGTTCCCAGGCTACCACGTA	AACCACGGTCTTTACTCCAC
seq-SEC61B-PBS15-F	CACCGGTAGTTCCCAGGCTACCACGTA	AACCACGGTCTTTACTCCAC
seq-SEC61B-PBS14-F	CACGATGAGTTCCCAGGCTACCACGTA	AACCACGGTCTTTACTCCAC
seq-SEC61B-PBS13-F	CGGAATCAGTTCCCAGGCTACCACGTA	AACCACGGTCTTTACTCCAC
seq-SEC61B-PBS12-F	GAGTGGAGTTCCCAGGCTACCACGTA	AACCACGGTCTTTACTCCAC
seq-SEC61B-RT17-F	GTGGCCAGTTCCCAGGCTACCACGTA	AACCACGGTCTTTACTCCAC
seq-SEC61B-RT16-F	TCCCGAAGTTCCCAGGCTACCACGTA	AACCACGGTCTTTACTCCAC
seq-SEC61B-RT15-F	GTCCGCAAGTTCCCAGGCTACCACGTA	AACCACGGTCTTTACTCCAC
seq-SEC61B-RT14-F	CGGAATCAGTTCCCAGGCTACCACGTA	AACCACGGTCTTTACTCCAC
seq-SEC61B-RT13-F	ACTGATGAGTTCCCAGGCTACCACGTA	AACCACGGTCTTTACTCCAC
seq-SEC61B-RT12-F	ATTCCTAGTTCCCAGGCTACCACGTA	AACCACGGTCTTTACTCCAC
seq-RUNX1-PBS16-F	ATGTCACGGTGAGGCTGAAACAGTGACC	TGCACCGAGGTGAAACAAGC
seq-RUNX1-PBS15-F	CGGAATCGTGAGGCTGAAACAGTGACC	TGCACCGAGGTGAAACAAGC
seq-RUNX1-PBS14-F	CACCGGTGTGAGGCTGAAACAGTGACC	TGCACCGAGGTGAAACAAGC
seq-RUNX1-PBS13-F	CACGATGGTGAGGCTGAAACAGTGACC	TGCACCGAGGTGAAACAAGC
seq-RUNX1-PBS12-F	GAGTGGGTGAGGCTGAAACAGTGACC	TGCACCGAGGTGAAACAAGC
seq-RUNX1-RT17-F	GTGGCCGTGAGGCTGAAACAGTGACC	TGCACCGAGGTGAAACAAGC
seq-RUNX1-RT16-F	TCCCGAGTGAGGCTGAAACAGTGACC	TGCACCGAGGTGAAACAAGC
seq-RUNX1-RT15-F	CGGAATCGTGAGGCTGAAACAGTGACC	TGCACCGAGGTGAAACAAGC

seq-RUNX1-RT14-F	GTCCGCAGTGAGGCTGAAACAGTGACC	TGCACCGAGGTGAAACAAGC
seq-RUNX1-RT13-F	ACTGATGGTGAGGCTGAAACAGTGACC	TGCACCGAGGTGAAACAAGC
seq-RUNX1-RT12-F	ATTCCTGTGAGGCTGAAACAGTGACC	TGCACCGAGGTGAAACAAGC
seq-SHANK3-PBS16-F	ATGTCACGCTAGCTGGTGAAGCGCCTTC	ACGTCCACCACAGGGCTCAG
seq-SHANK3-PBS15-F	CACCGGTCTAGCTGGTGAAGCGCCTTC	ACGTCCACCACAGGGCTCAG
seq-SHANK3-PBS14-F	CACGATGCTAGCTGGTGAAGCGCCTTC	ACGTCCACCACAGGGCTCAG
seq-SHANK3-PBS13-F	CGGAATCCTAGCTGGTGAAGCGCCTTC	ACGTCCACCACAGGGCTCAG
seq-SHANK3-PBS12-F	GAGTGGCTAGCTGGTGAAGCGCCTTC	ACGTCCACCACAGGGCTCAG
seq-SHANK3-RT17-F	GTGGCCCTAGCTGGTGAAGCGCCTTC	ACGTCCACCACAGGGCTCAG
seq-SHANK3-RT16-F	TCCCGACTAGCTGGTGAAGCGCCTTC	ACGTCCACCACAGGGCTCAG
seq-SHANK3-RT15-F	GTCCGCACTAGCTGGTGAAGCGCCTTC	ACGTCCACCACAGGGCTCAG
seq-SHANK3-RT14-F	ACTGATGCTAGCTGGTGAAGCGCCTTC	ACGTCCACCACAGGGCTCAG
seq-SHANK3-RT13-F	CGGAATCCTAGCTGGTGAAGCGCCTTC	ACGTCCACCACAGGGCTCAG
seq-SHANK3-RT12-F	ATTCCTCTAGCTGGTGAAGCGCCTTC	ACGTCCACCACAGGGCTCAG
seq-HEK3-WT-F	ATCACGAGGGAAACGCCCATGCAAT	CCTCCCTAGGTGCTGGCTT
seq-HEK3-G4-F	CGATGTTAGGGAAACGCCCATGCAAT	CCTCCCTAGGTGCTGGCTT
seq-HEK3-evopreQ1-F	TTAGGCAGAGGGAAACGCCCATGCAAT	CCTCCCTAGGTGCTGGCTT
seq-RNF2-WT-F	ATCACGAGCCAACATACAGAAGTCAG	TTTCCAGCAATGTCTCAGGC
seq-RNF2-G4-F	CGATGTTAGCCAACATACAGAAGTCAG	TTTCCAGCAATGTCTCAGGC
seq-RNF2-evopreQ1-F	TTAGGCAGAGCCAACATACAGAAGTCAG	TTTCCAGCAATGTCTCAGGC
seq-VEGFA-WT-F	ATCACGACTTGGTGCCAAATTCTTCTCC	AAGAGGGAATGGGCTTTGGA
seq-VEGFA-G4-F	CGATGTTACTTGGTGCCAAATTCTTCTCC	AAGAGGGAATGGGCTTTGGA
seq-VEGFA-evopreQ1-F	TTAGGCAGACTTGGTGCCAAATTCTTCTC	AAGAGGGAATGGGCTTTGGA
seq-DNMT1-WT-F	ATCACGCACAACAGCTTCATGTCAGC	GGTCCATGTCTGTTACTCGC
seq-DNMT1-G4-F	CGATGTTCACAACAGCTTCATGTCAGC	GGTCCATGTCTGTTACTCGC
seq-DNMT1-evopreQ1-F	TTAGGCAGCACAACAGCTTCATGTCAGC	GGTCCATGTCTGTTACTCGC
seq-RUNX1-WT-F	ATCACGAAGAAAGAGAGATGTAGGGC	CATTACAGGCAAAGCTGAGC
seq-RUNX1-G4-F	CGATGTTAAGAAAGAGAGATGTAGGGC	CATTACAGGCAAAGCTGAGC
seq-RUNX1-evopreQ1-F	TTAGGCAGAAGAAAGAGAGATGTAGGGC	CATTACAGGCAAAGCTGAGC
seq-LSP-G4-F	AGTTCCACTCAAGAGGTCCCCAAATGC	CCATCACACAGGGAGGAGGT
seq-LSP-evopreQ1-F	ATGTCACGCTCAAGAGGTCCCCAAATGC	CCATCACACAGGGAGGAGGT
seq-LSP-Mpknot-F	CACCGGTCTCAAGAGGTCCCCAAATGC	CCATCACACAGGGAGGAGGT
seq-LSP-XR-F	CACGATGCTCAAGAGGTCCCCAAATGC	CCATCACACAGGGAGGAGGT

seq-FANCF-G4-F	AGTTCCAGGATGTTCCAATCAGTACGC	ATGCCGACCAAAGCGCCGAT
seq-FANCF-evopreQ1-F	ATGTCACGGGATGTTCCAATCAGTACGC	ATGCCGACCAAAGCGCCGAT
seq-FANCF-Mpknot-F	CACCGGTGGATGTTCCAATCAGTACGC	ATGCCGACCAAAGCGCCGAT
seq-FANCF-XR-F	CACGATGGGATGTTCCAATCAGTACGC	ATGCCGACCAAAGCGCCGAT
seq-SEC-G4-F	AGTTCCAAGTTCCCAGGCTACCACGTA	AACCACGGTCTTTACTCCAC
seq-SEC-evopreQ1-F	ATGTCACGAGTTCCCAGGCTACCACGTA	AACCACGGTCTTTACTCCAC
seq-SEC-Mpknot-F	CACCGGTAGTTCCCAGGCTACCACGTA	AACCACGGTCTTTACTCCAC
seq-SEC-XR-F	CACGATGAGTTCCCAGGCTACCACGTA	AACCACGGTCTTTACTCCAC
seq-UBE3A-G4-F	AGTCAATCATAGAGACAACTCTGGGT	TTCTGGCCACTCTTATATTC
seq-UBE3A-evopreQ1-F	AGTTCCATCATAGAGACAACTCTGGGT	TTCTGGCCACTCTTATATTC
seq-UBE3A-Mpknot-F	ATGTCACGTCATAGAGACAACTCTGGGT	TTCTGGCCACTCTTATATTC
seq-UBE3A-XR-F	CACCGGTTCATAGAGACAACTCTGGGT	TTCTGGCCACTCTTATATTC

Supplementary Table S4. Primers used for off-target analysis.

NO.	Forward Primer	Reverse Primer
seq-SEC61B-peg-OT1	CATCAGTGCAGACTTCTAGC	GTTTGGCCAAATCAAAGGAT
seq-SEC61B-peg-OT2	TGCAGCCCCTCTATACCAAT	TAAAGTACACACATTCAGAC
seq-SEC61B-peg-OT3	CAGTCAGATTTTTGGCCTAG	CACACTGGCTCTCAGTGAAG
seq-SEC61B-peg-OT4	GAGTAGCCTTCCAAAGGGAA	ATGTTGCCCAAAGGAAAAGA
seq-SEC61B-peg-OT5	CGCCACTGATTCATCCTTTG	CATGCAAGTGGGTATTATTT
seq-SEC61B-nick-OT1	CTGCTGTGACAGGGCTGCAG	CTCAGGTGAGCAATCACAGT
seq-SEC61B-nick-OT2	CCAGGGGCATGTTGTAGGGC	TCCTGCTCTGCCCCTGTGGC
seq-SEC61B-nick-OT3	GATGAACACAATGGAGAGGA	GTACCTTGAGTTTGGAAAGA
seq-SEC61B-nick-OT4	GGACCTATGCTGTTCCCAAG	GGCAAGATACAGGGCAGGAC
seq-SEC61B-nick-OT5	ATGCAGCTGAAGACCTGGTA	TTGGGTTCCTCGTCTGCTAC
seq-SEC61B-nick-OT6	ATGCCAGGCCCCATAATATT	AAGTGGCCCAGATGGTCGAA
seq-RNF2-peg-OT1	GCTGTGCCACCCACACACCA	TGACCTAAAAGGCCCTCACA
seq-RNF2-peg-OT2	AGTCAGGAGCTTGCAGCACA	CCATGTGTGGCTGTCAAGCA
seq-RNF2-peg-OT3	ACCAGGTATCTGTCTTGACA	CCTATCTTTACAGCACAGGT
seq-RNF2-peg-OT4	TTGGGCAGGGACACACATCC	GATGAGGAACCAAGGCATCC
seq-RNF2-peg-OT5	CCCAGAAAGCTTCCTTACCT	GTTATATGCAGCCATTGTGG
seq-RNF2-nick-OT1	CAGCACAGTGATTTTGAGAG	ATAAGGCACTGCAATCAATC
seq-RNF2-nick-OT2	AGACAGGCTCTGAGTACACT	AGCAAGAGGCAAGGACCATG
seq-RNF2-nick-OT3	CAGTGAAGTGAGTGGGTGGG	GCCAGCTCACAAGTCTGGAG
seq-RNF2-nick-OT4	GTGATGACAGAGGTGCCGGT	TCCCAAAGTGCTGGGATTAC
seq-RUNX1-peg-OT1	TCTGGCAAAGGGGATCGTGG	TGGTTTCCTCAGCTTAGGGC
seq-RUNX1-peg-OT2	GCATGAGGGGTACTGGCCTT	TGTCGGGTGGTCTGATAGCT
seq-RUNX1-peg-OT3	CATGGGTAAATGCAATACCC	GTGCCTGTCTGATCTGAATA
seq-RUNX1-peg-OT4	CCACTGTATTCCAGGTGCCT	TTCCAGGGCTAGCGGGTGAT
seq-RUNX1-peg-OT5	CTTATGGTGAACTGCCAGGG	CCCATTGTTCTGGCTGTTAG
seq-RUNX1-nick-OT1	GCAAGCAGCCCGCCACCATC	GAAGAGACACTAGCTACTCG
seq-RUNX1-nick-OT2	CAGACCTTACAACTGAAGCT	GATCCCACGCATTTGAAACA
seq-RUNX1-nick-OT3	GAACCCTGAGGACATTTTGC	TCATCGTGCAGGCACCACCA
seq-RUNX1-nick-OT4	GTCATCTTCCTGGTTGAGCT	ACCAGCAGCTGGGATGCAGA
seq-RUNX1-nick-OT5	CCAGACCATCCATGGCTGTC	GACCCTGCACAGAGCAACAA
